# Effect of different constraining boundary conditions on simulated femoral stresses and strains during gait

**DOI:** 10.1038/s41598-024-61305-x

**Published:** 2024-05-11

**Authors:** Alireza Y. Bavil, Emmanuel Eghan-Acquah, Laura E. Diamond, Rod Barrett, Christopher P. Carty, Martina Barzan, Azadeh Nasseri, David G. Lloyd, David J. Saxby, Stefanie Feih

**Affiliations:** 1 Griffith Centre of Biomedical and Rehabilitation Engineering (GCORE), Griffith University, Gold Coast, Australia; 2 School of Health Sciences and Social Work, Griffith University, Gold Coast, Australia; 3 Advanced Design and Prototyping Technologies (ADaPT) Institute, Griffith University, Gold Coast, Australia; 4 School of Engineering and Built Environment, Griffith University, Gold Coast, Australia

**Keywords:** Bone mechanics, Boundary conditions, Finite element analysis, Neuromusculoskeletal modelling, Orthopaedics, Bone, Biomedical engineering, Paediatric research

## Abstract

Finite element analysis (FEA) is commonly used in orthopaedic research to estimate localised tissue stresses and strains. A variety of boundary conditions have been proposed for isolated femur analysis, but it remains unclear how these assumed constraints influence FEA predictions of bone biomechanics. This study compared the femoral head deflection (FHD), stresses, and strains elicited under four commonly used boundary conditions (fixed knee, mid-shaft constraint, springs, and isostatic methods) and benchmarked these mechanics against the gold standard inertia relief method for normal and pathological femurs (extreme anteversion and retroversion, coxa vara, and coxa valga). Simulations were performed for the stance phase of walking with the applied femoral loading determined from patient-specific neuromusculoskeletal models. Due to unrealistic biomechanics observed for the commonly used boundary conditions, we propose a novel biomechanical constraint method to generate physiological femur biomechanics. The biomechanical method yielded FHD (< 1 mm), strains (approaching 1000 µε), and stresses (< 60 MPa), which were consistent with physiological observations and similar to predictions from the inertia relief method (average coefficient of determination = 0.97, average normalized root mean square error = 0.17). Our results highlight the superior performance of the biomechanical method compared to current methods of constraint for  both healthy and pathological femurs.

## Introduction

The emergence of in silico analysis has notably advanced clinical decision-making in orthopaedics, with finite element analysis (FEA) playing a pivotal role. FEA offers profound insights into biomechanics across various scenarios, proving crucial for fracture risk assessment in metastatic bone disease^1^, orthopaedic implant design, evaluation, and optimization^2^, comprehending the biomechanical effects of osteoporosis and osteoarthritis^3^, and allowing for more customized patient treatment strategies^4^. Crucially, FEA enables the study of important tissue-level localised stresses and/or strains that cannot be measured in vivo. Studies implementing FEA have presented results for stresses and strains in bone^5–11^, stresses in implants^12–19^, micromotions between bone and implant^20–23^, bone remodelling^24–27^, and stress shielding^28–32^ in response to a range of applied loading conditions. Nonetheless, the efficacy of FEA is critically dependent on the accurate determination of boundary conditions—a challenge that has not been comprehensively addressed in femoral FEA studies^33–35^. This underpins our current study, which aims to assess and contrast the boundary conditions commonly cited in existing literature. Our objective is to identify advocate for an established or innovative boundary condition that enhances the predictive precision and practical application of FEA in femoral orthopaedic research.

To create a determinate solution to any quasi-static FEA problem for an isolated tissue-level system, the model must be adequately constrained in the form of displacements and/or rotations to prevent rigid body motions and maintain static equilibrium. Stresses and strains in such an isolated bone tissue system can be significantly over-predicted if the system is over-constrained. Despite the widespread use of FEA within the biomechanics community, the lack of standardization of adequate constraining boundary conditions limits confidence in model predictions and makes comparisons of tissue stresses and strains between studies challenging. In addition, constraining boundary conditions will depend on the isolated tissue system to be studied.

Pre-empting our results, we undertook a systematic review of peer-reviewed literature to identify the various boundary conditions applied to FEA models of isolated femurs (see Supplementary Material S1). It was found that research groups have generally used five main groupings of boundary conditions, which we labelled as (i) *fixed knee*, (ii) *springs*, (iii) *mid-shaft constraint*, (iv) *isostatic constraint*, and (v) *inertia relief (IR)* methods. The *fixed knee* and *mid-shaft* methods rigidly fix the distal femur and mid-diaphysis in all degrees of freedom (DoF), respectively. The *springs method* uses multiple weak springs for support, and the *isostatic* method applies constraints to three distinct femoral regions. The non-physiological nature of these boundary conditions may result in over or underestimation of femoral bone mechanics constraints. To date, no robust analysis of the effects of these boundary conditions on simulated bone mechanics has been performed. A rigorous assessment of best numerical practice is critical, as individual studies have shown internal stress states of the femur (and likewise other isolated organs) to be highly sensitive to applied forces and boundary conditions^33–35^.

In contrast to the above listed boundary conditions, the *IR* method^36^ enables accurate numerical solutions based on static force equilibrium without displacement constraints. The* IR* method is considered the best-practice approach for an isolated system, as it establishes an applied force and moment balance without the need for any additional constraints^37–39^. Remaining residual forces, which generally arise during neuromusculoskeletal (NMSK) simulations of bone loading because the human skeletal system solution is iteratively derived during dynamic human locomotion, are then accounted for by applying equivalent accelerations to the FEA model of the isolated tissue-level system. Surprisingly, this method has gained little traction within the FEA research community, which may be because it is not widely implemented in popular open-access research software (e.g., FEBio). Furthermore, some commercial FEA software packages, such as Abaqus^40^, do not support use of the *IR* method in multi-component models with contact formulation between adjacent surfaces (e.g., simulation of bone-implant mechanics). The limitations inherent in the *IR* method, coupled with the potential inadequacies of frequently employed boundary conditions, necessitate the present in-depth study and the development of a novel approach that not only ensures biomechanical accuracy but also facilitates seamless integration across diverse modelling scenarios.

To benchmark FEA outputs, three key experimental measures are reported in the literature in FEA studies of femoral stresses and strains. First, the femoral head deflection (FHD) provide insights into the displacements and deformations of the femoral head, which are crucial for understanding joint contact mechanics and load transfer^41^. Second, the peak von Mises stress (PVMS) is a critical measure for evaluating the mechanical integrity of the femur, as it indicates regions of high stress concentration that could potentially lead to failure or structural damage^42^. Third, cortical strains offer valuable information about the deformation and structural response of the femur's outer cortical bone, aiding in characterization of a bone’s mechanical properties and assessment of potential tissue-level adaptations^35,41^. Furthermore, since femoral stress and strain magnitudes during gait are, in part, a function of femoral geometry^43^, and because there can be significant variation in femoral geometry in conditions such as *coxa vara/valga*, femoral anteversion and retroversion, it is important to evaluate the effects of different boundary condition formulations on model predictions across a broad spectrum of femoral geometries.

The purpose of this study is, therefore, to compare the predicted and experimental FHD, PVMS, and cortical strains observed during the stance phase of gait when applying commonly used boundary condition methods. These boundary conditions comprise a new biomechanical approach based on physical femur motion, compared to the *IR* method, in normal and pathological femurs (*coxa vara*, *coxa valga*, and severe anteversion and retroversion). All the forces applied to different FEA models were computed from personalised gait data for a representative participant using an NMSK model. We highlight that the newly developed biomechanical method results in closer agreement with the *IR* method than the other aforementioned methods reported in the literature.

## Methods

### Selection of boundary conditions

Briefly, we undertook a systematic review of different approaches to specifying, or defining, the boundary conditions used when predicting the stress state of an isolated femur (see Supplementary Material S1 for a full description). We found numerous approaches to setting boundary conditions (Supplementary Material Table S1), which all had certain limitations that are now briefly described.

Typically, articulating surfaces of the femoral condyles were fully constrained in all translational and rotational DoF, a simplistic representation of the knee joint that is better represented as a moving hinge-type joint with both rotational and sliding motion^44^. Several studies also constrained either femur diaphysis or head^45–47^ in all DoF. Again, these constraints do not mimic the physiological mechanics of the human femur as the effects of distally attached muscles, as well as tibiofemoral and patellofemoral contact are minimised, while constraints also do not respect the dynamics of the NMSK system used to calculate joint and muscle forces.

To avoid the limitation of using fixed boundary conditions, some studies used a free boundary condition approach. Herein muscles and ligaments were explicitly modelled as spring elements while three-dimensional objects represented the acetabulum and tibial plateau^48–51^. Similarly, some studies attempted to model physiological boundary conditions using minimal isostatic constraints (Supplementary Material, Table S1), which aimed to mimic natural femoral head deflections, while also minimizing errors due to unrealistic modelling assumptions of conventional methods. Typically, the *isostatic* method involves constraining three translational DoF of a model node at the knee center, two translational DoFs of the node at the location of hip contact (such that this node can only deflect along the femur mechanical axis), and one DoF of a node on the lateral epicondyle in anterior–posterior direction^35^. Counterintuitively, this approach has the limitation of directing the deformation of the femur head along the femur’s mechanical axis, but does not account for rotational and anterior–posterior motions of the femur and femoral head during gait^52^.

**Figure 1:**
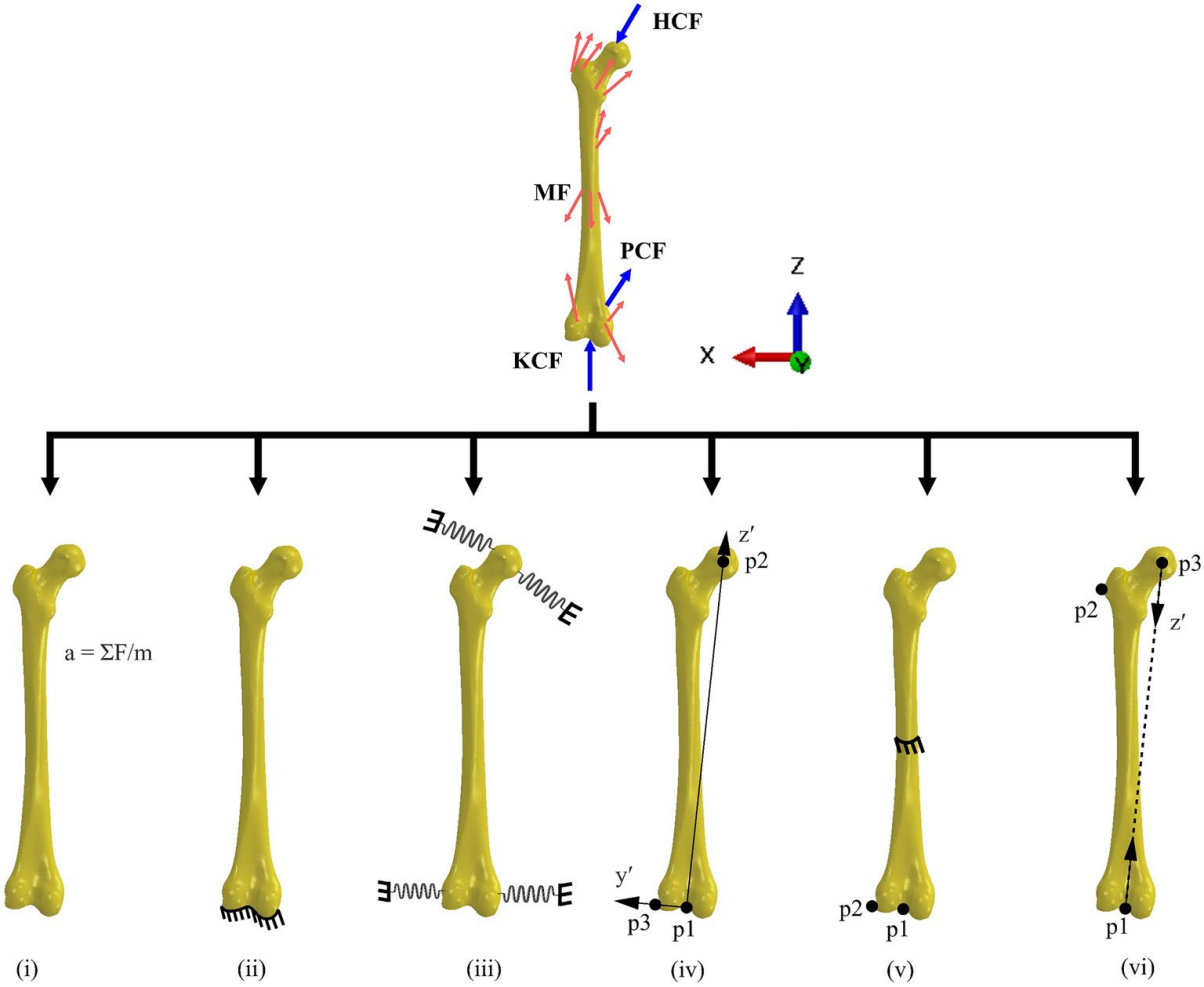
The six boundary conditions applied to a right femur throughout stance phase of gait. (**i**) *Iinertia relief*, (**ii**) *fixed knee*, (**iii**) *springs*, (**iv**) *isostatic constraint*, (**v**) *mid-shaft constraint*, and (**vi**) *biomechanical constraint*.

The systematic review (Supplementary Material S1) resulted in five distinct sets of boundary conditions that were used in full-femur models to which we added a sixth boundary condition, and all six were compared in this study. Boundary condition selections for FEA studies were limited to those explored for full-femur model simulations. The sixth boundary condition was a method we developed to address the abovementioned limitations. Each condition had a unique set of spatial displacement and/or force/moment constraints for the isolated femur that resulted in a unique mathematical solution of the FEA problem (Fig. 1). For all boundary conditions, muscle forces were applied to the same reference points coupled to surfaces representing average muscle attachment areas, respectively. A reference point positioned at the femur head’s center was coupled to a nodal region on the femur head cortex, which serves as the articulating surface between the femur and the pelvis. This reference point was determined by fitting a sphere to the femur head and designating the sphere’s center as the femur head center. Unless otherwise specified, the hip contact force was applied to this reference point. The six boundary conditions evaluated were as follows:

- *Inertia relief (IR)* This method allows FEA of the femur without setting arbitrary displacement and rotation constraints leading to additional reaction forces due to artificial over-constraining^36,40^. The femur is assumed to be in state of (dynamic) equilibrium, but *IR* neutralizes the effects of residual forces causing femoral motion by applying equivalent inertial loads to place the femur in quasi-static equilibrium loading conditions^36,40^. All muscle and joint contact forces, including tibiofemoral and patellofemoral, were applied to the model (Fig. 1i).
- *Fixed knee* This method has been commonly used in most FEA of the isolated femur. Specifically, this method constrains the distal femur in all six DoF, with subsequent application of hip contact force and targeted muscle forces, while neglecting the potential influence of knee and patellofemoral contact forces (Fig. 1ii).
- *Springs* In this method, the femur is not constrained. Rather, multiple weak springs are affixed to hip and knee joints to prevent rigid motion of the femur. The spring sets are attached to groups of nodes surrounding the femoral neck shaft, epicondyles, and knee joint center. The inclusion of numerous weak springs affixed to multiple nodes in the region of interest on the outer surface of femur aims to avert stress concentrations on individual elements. Consequently, all muscle and contact forces are applied in the model (Fig. 1iii).
- *Isostatic constraint* All muscle and contact forces are considered and applied at their respective anatomical locations. A local coordinate system is defined such that the superior-inferior axis is formed from the centers of the femoral head and knee, the medial–lateral axis is formed from the knee center to lateral epicondyle, and the anterior–posterior axis is the cross-product of medial–lateral and superior–inferior axes. All translational DoF are fixed for the node located at knee center. Anterior–posterior and medial–lateral DoF for center of femur head are also fixed, and the superior–inferior DoF is fixed for nodes at lateral epicondyle^35^ (Fig. 1iv).
- *Mid-shaft constraint* All muscle and joint contact forces are applied at their respective locations. The knee joint center is constrained in all three translational DoF, the lateral epicondyle constrained in anterior–posterior DoF, and the mid-diaphysis of the femur constrained in the medial–lateral and anterior–posterior DoF (Fig. 1v).
- *Biomechanical constraint* Like boundary conditions (i)–(v), the new biomechanical constraint method incorporated all muscle and joint contact forces. A local coordinate system, analogous to the *isostatic* method, was established, and an axial translational connector was defined between femur head center and knee center. The hip contact force is then applied through the defined connector (p1–p3), thereby allowing the femur head to translate and rotate without imposition of artificial constraints. This approach effectively mimics the role of the acetabulum in femur mechanics. The knee joint center is fixed in all translational DoF. Additionally, to ensure anatomical accuracy, the most lateral region (p2 in Fig. 1vi) of the greater trochanter is constrained in medial–lateral and anterior–posterior DoF, as well as the rotation around superior–inferior axis, to simulate the passive constraining effects of thigh soft tissues (Fig. 1vi).

### Participant information and computed tomography imaging

Medical imaging data from an 11-year-old female (height = 1.72 m; weight = 93.4 kg) who participated in a medical device trial at our institution was used for this study. As part of surgical planning for a unilateral proximal femoral deformity, the participant underwent bilateral full femur low dose CT scans and preoperative gait analysis. Therefore, a full femur CT of typical geometry was available, leaving room for later modifications to create pathological femurs. These scans were collected according to the clinical management protocol and de-identified and shared for this FE analysis. Ethics approval for the medical device trial and the secondary analysis of clinical medical imaging data was approved from the institutional ethics committee (HREC/18/QRCH/161—Children’s Health Queensland Hospital and Health Service Human Research Ethics Committee; GU Ref No: 2019/770) and written informed consent to participate in the trial was obtained from the participants. All procedures were performed in accordance with relevant guidelines and regulations (principles set by the Declaration of Helsinki). The CT acquisition sequence of the scanner (Somatom force syngo VB10A; SIEMENS, Munich, Germany) resulted in 1.5 mm slice thickness and 1 mm interslice distance. Biomechanical data acquisition and processing.

The participant underwent a comprehensive gait analysis at the Queensland Children’s Motion Analysis Service (QCMAS), Center for Children’s Health Research (Brisbane, Australia). During this gait analysis, retroreflective markers were placed atop key anatomical landmarks following recommendations of Kainz et al.^53^. A calibrated 10-camera motion capture system (Vicon Motion Systems Ltd, UK) was used to collect participant motion during a series of static and walking trials performed at a self-selected pace. At least one complete stance phase of the right leg was recorded for the walking trials. Four force platforms (510 mm × 465 mm, AMTI, Watertown, MA, USA) measured ground reaction forces (GRF) at 1500 Hz. Wireless surface electromyography (EMG) sensors (Noraxon, AZ, USA) were secured over 12 muscles of the right leg: gluteus maximus and medius, semitendinosus, biceps femoris long head, rectus femoris, vastus medialis and lateralis, gastrocnemius, tibialis anterior, gracilis, tensor fasciae latae, and sartorius. EMG signals were recorded at 1000 Hz, and surface electrode placement was in accordance with the Surface Electromyography for the Non-Invasive Assessment of Muscles (SENIAM) guidelines^54^.

### Neuromusculoskeletal modelling

The open-source MOtoNMS toolbox^55^ in MATLAB (Mathworks, MASS, USA) was used to convert raw EMG, GRF, and marker trajectories from a coordinate 3D file (c3d) to file formats and data conventions used by the open-source biomechanical software OpenSim^56^. EMG Linear envelopes were generated using sequentially applied signal conditioning methods: (1) a dual-pass 2nd-order high-pass Butterworth filter with a cut-off frequency of 30 Hz was employed, (2) full-wave rectification, (3) a dual-pass 2nd-order low-pass Butterworth filter with a nominal cut-off frequency of 6 Hz. The resulting EMG linear envelopes were then amplitude-normalized to each muscle’s maximum amplitude and henceforth referred to as muscle excitations. Marker trajectories and GRF were filtered with a 4th-order (zero-lag) Butterworth filter with a cut-off frequency of 6 Hz^57,58^.

To model the external biomechanics of the participant, the Rajagopal 2015 model^59^ was used in OpenSim v3.3^56^. This model consisted of 37 DoF and 80 muscle–tendon units (MTU) actuating the lower limb. The model was first linearly scaled to match the anthropometry of the patient using marker positions from the static trial^53^. The optimal fiber and tendon slack lengths of the MTU of the lower limbs were then optimized to preserve their dimensionless force–length operating curves across a physiological and multi-DoF range of motion^60^. Each muscle’s maximum isometric forces were then personalized based on the participant’s mass and height^61^.

To solve for the model’s joint rotations and translations (i.e., generalized coordinates), inverse kinematics was performed which mobilizes the general coordinates of the scaled model such that the least squares error is minimized between the experimental markers and their corresponding virtual markers on the model. To solve for generalized loads (i.e., net joint moments and forces), an inverse dynamics solution was derived by combining the external loads applied to the body (i.e., GRF) with the model (with mass and inertia) and motions (from inverse kinematics) to resolve the Newton–Euler equations. Last, the kinematics of each MTU (i.e., moment arms, lengths, and lines of action^62^) were computed using the Muscle Analysis tool. Collectively, we refer to the generalized coordinates, generalized loads, and MTU kinematics as the external biomechanics as they do not involve modelling any internal physiological processes such as contraction dynamics and can be resolved from external observations only.

To estimate the internal biomechanics (e.g., muscle activations, muscle forces, and, subsequently, joint contact forces), the Calibrated EMG-Informed NeuroMusculoSkeletal (CEINMS) toolbox was used. This toolbox makes use of experimental measures of muscle excitation (e.g., normalized linear envelopes from EMG) to predict muscle forces that satisfy the generalized loads within the model determined by inverse dynamics^63,64^. The EMG-assisted neural mode within CEINMS was selected for this study^65^ as it provides an excellent compromise between generating muscle-driven joint torques that match those from inverse dynamics while also respecting the experimentally acquired measures of muscle excitation (i.e., EMG). This neural solution mode has been used numerous times to compute joint contact forces at the hip^66–68^, knee^69,70^ and ankle^68^.

First, NMSK model parameters were calibrated within physiological bounds. Parameters included those governing MTU mechanics (i.e., optimal fiber and tendon slack lengths, strength coefficients) and muscle excitation-to-activation dynamics. Calibration was performed using three walking trials (Table 1). Inverse dynamics, inverse kinematics, MTU kinematics and 12 experimental muscle excitations mapped across 23 MTU were used for subsequent CEINMS execution in EMG-assisted mode (see Supplementary Material, Table S2).

**Table 1:** The range of neuromusculoskeletal parameters selected for calibration.

Neuromusculoskeletal parameter	Range	
	Minimum	Maximum
Tendon slack length	0.95	1.25
Optimal fiber length	0.95	1.50
Muscle strength coefficient	0.5	1.05
Non-linear shape factor	− 4.999	− 0.001
MTU activation filter coefficients	− 0.95	− 0.05

*MTU* muscle–tendon unit.

The calculation of the muscle line of action^62^ from the muscle analysis (above) decomposed the orientation of each muscle action into its three-dimensional components. Decomposed lines of action as well as three-dimensional hip, tibiofemoral, and patellofemoral joint contact forces were transformed from the global to the local femoral coordinate system using custom MATLAB scripts. The magnitudes of these loads across one stance phase of the gait cycle were discretised into seven equally spaced instances and subsequently used as loading conditions in seven quasi-static nonlinear analysis steps for the femur FEA.

### Sequentially linked neuromusculoskeletal-finite element analysis

**Figure 2:**
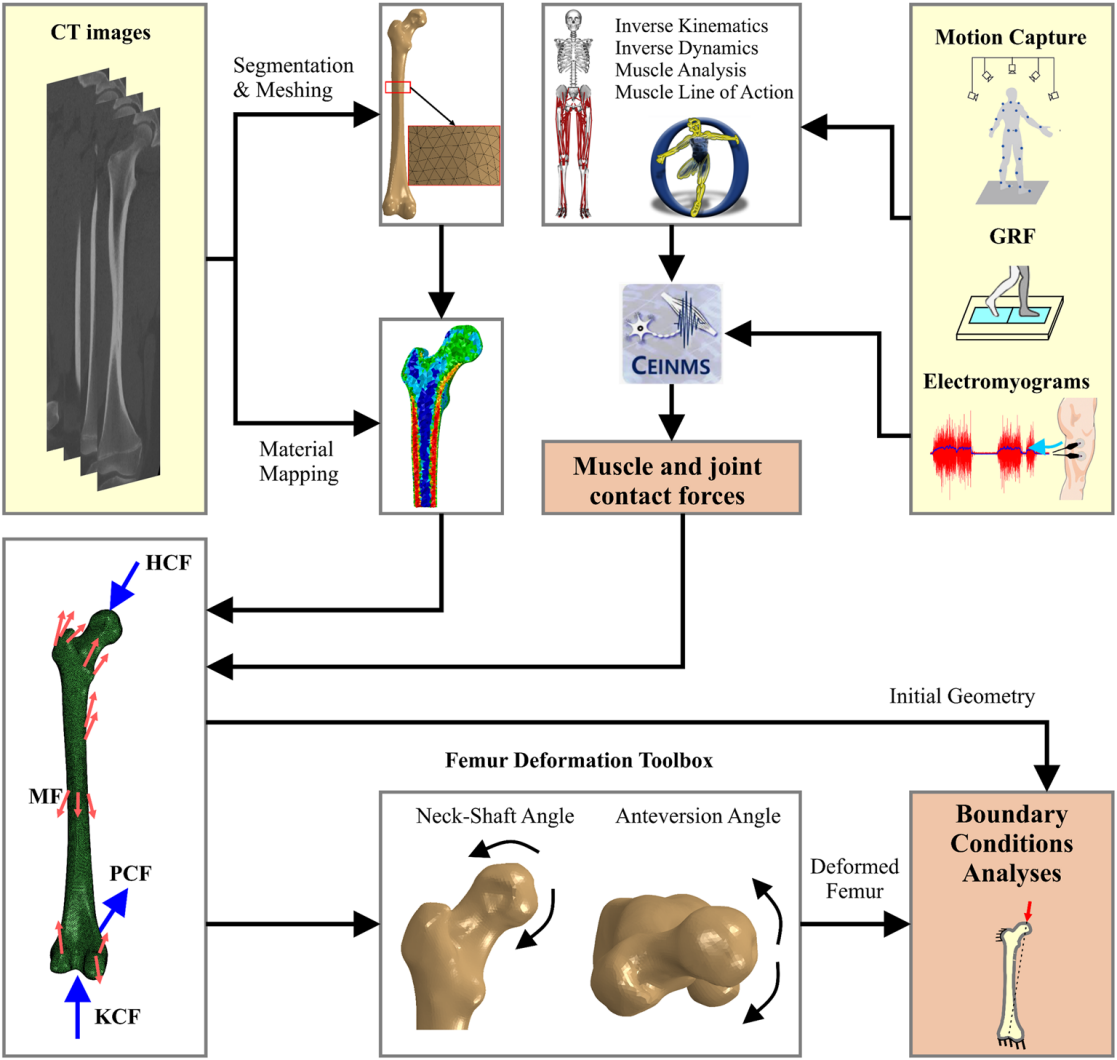
The workflow of the NMSK-FEA process. The participant’s computed tomography (CT) data of the isolated femur were segmented, meshed, and assigned material properties. Model motion, net loading, and MTU kinematics, along with muscle excitations from walking gait were input to CEINMS to estimate muscle and joint contact forces. The muscle and joint contact forces were applied to the FEA model under various boundary conditions to simulate the mechanics of the isolated femur.

The summary of the NMSK-FEA pipeline is presented (Fig. 2). The personalized FEA model was created using medical imaging described in “Participant information and computed tomography imaging” section and respective material mapping. Abaqus Simulia v2022 (Dassault Systèmes, Simulia Corp, United States) was used for the nonlinear quasi-static analysis. Muscle attachment points were modelled as reference points in Abaqus located in the respective positions on the femur body in the scaled OpenSim model. These reference points were coupled to representative regions on the outer femur surface to mimic the anatomical connection of muscles to a finite surface area of the bone, thereby preventing unrealistic stress concentrations within these regions. For each model and loading condition, the six different approaches to boundary conditions were imposed, and simulations were confirmed to converge with mesh refinement.

#### Medical image processing and material mapping

The participant’s right femur was segmented using Mimics v25 (Materialise NV, Leuven, Belgium). The femur’s geometry was approximated based on greyscale value (GV) with a threshold range of 1250 to 4095, which was selected to best represent the average bone mineral density^71^. From this segmentation, cavities were filled using three-dimensional interpolation, followed by wrapping and smoothing to close any remaining small cavities and improve surface smoothness for meshing.

The resulting three-dimensional femur model was volume-meshed using Hypermesh v14.0 (Altair Engineering, Inc, Troy MI, United States). An average surface mesh size of 2 mm was deemed suitable for this analysis, as established by prior mesh convergence studies (see Supplementary Material, Fig. S2). Meshing the femur model with an average seeding size of 2 mm resulted in a highly detailed femur model consisting of 47,511 nodes and 229,779 linear tetrahedral (C3D4) elements capturing the cortical and cancellous structures of the bone. In-house analyses confirmed that the C3D4 element type performed similarly to other element types for the selected evaluation criteria and was selected due its computational efficiency.

The femur model was assigned participant-specific material properties using established material mapping equations (see Eqs. (1), (2)),

1 $$\begin{array}{c}{\rho }_{app}=0.001029GV+0.114259\, {\text{g}}\text{/}{\text{cm}}^{3},\end{array}$$

2 $$\begin{array}{c}E=6850{{\rho }_{app}}^{1.49} {\text{ MPa}},\end{array}$$

where ${\rho }_{app}$ is the apparent bone density, and *E,* is the Young’s modulus^72–74^.

A sensitivity study of the material property sets was conducted to ensure results were independent of the number of material property sets applied to the model (Supplementary Material, Fig. S3). The results of this sensitivity analysis indicated ten sets of material properties were appropriate, and these were subsequently mapped to the femur model. The Poisson’s ratio for each element (irrespective of the mapped modulus) was set ν = 0.3^75^.

#### Femur deformation toolbox

**Figure 3:**
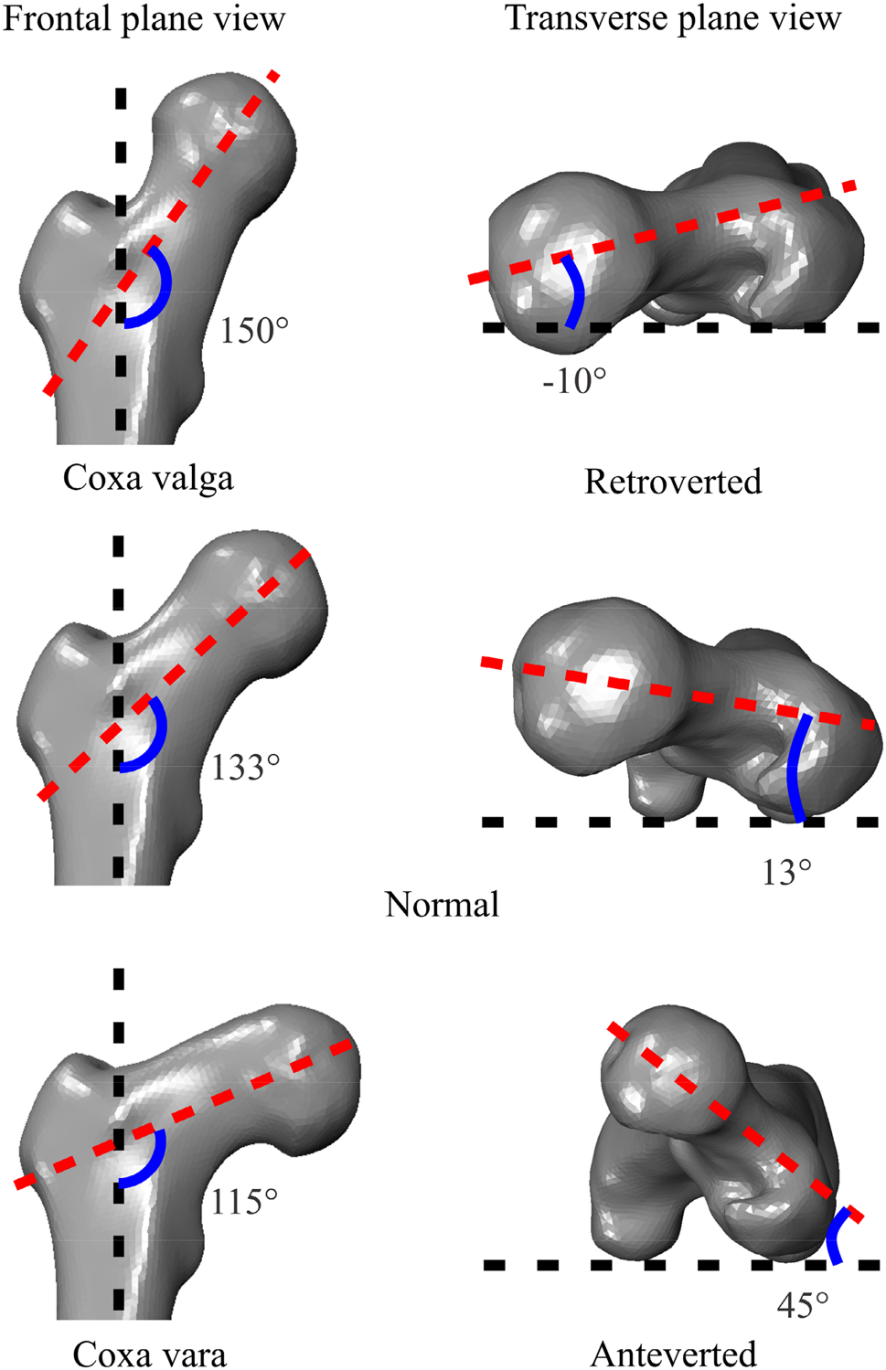
Normal (middle row), retroverted (top right), anteverted (bottom right), *coxa valga* (top left), and *coxa vara* (bottom left) femur geometries.These geometries were implemented, and the effect of boundary conditions on femur mechanics was subsequently assessed.

The effects of boundary conditions on isolated femur mechanics were also considered for femurs with deformities, which are potential candidates for osteotomy. To impart deformities, a MATLAB toolbox was designed to gradually apply the prescribed torsion to the femur while preserving mesh quality and property definition. Anteversion angles (AVA) of 45° and -10°^76^ were achieved using this toolbox by gradually rotating the femoral shaft of the FEA model from its original (neutral) alignment. Additionally, neck-shaft angles of 150° and 115°, which represent *coxa valga* and *coxa vara*^77,78^, respectively, were constructed by gradually rotating the femur neck shaft (Fig. 3). This femur deformation toolbox circumvented the need for reconstructing FEA models of the various femur deformities and facilitated rapid implementation (i.e., execution in a few seconds).

### Outcome measures

#### Femoral head deflection

The FHD describes the general deformation of the bone and has been measured previously^41^. The FHD is a critical mechanical parameter, and indirectly reflects bending loads exerted on the femur. An accurate estimation of the FHD is crucial to avoid misjudgements in implant design and surgical planning. Under- or overestimation of the FHD can lead to erroneous conclusions and potentially compromise the success of surgical interventions. Ranges of 0 mm to 3 mm in superior–inferior and -1.5 mm to 4 mm in medial–lateral directions for the FHD are considered physiologically reasonable for single-legged stance^41^. The FHD was assessed in all anatomical directions for each load step by calculating displacement of the hip joint center relative to the knee joint contact center for all models and compared to corresponding simulations using the *IR* method. To calculate the FHD, hip and knee joint centres were selected, and their 3-dimensional displacement was calculated throughout the analysis. An in-house python script was used to perform the vector calculations to determine the relative displacement of hip joint center with respect to knee joint center.

#### Peak von Mises stress

The PVMS provides insight into potential failure of bone segments and implants. All models were compared to the *IR *method. The coefficient of determination (R^2^) and root mean square error normalised to the dynamic range (nRMSE) were calculated for normal and pathological femur geometries.

#### Cortical strain

The pattern and magnitude of the cortical strain are important parameters to assess deformations in the femoral shaft^41^. The strains on the femur diaphysis were evaluated along predefined paths on medial, lateral, anterior, and posterior cortical aspects.

## Results

### Femoral head deflections

**Figure 4:**
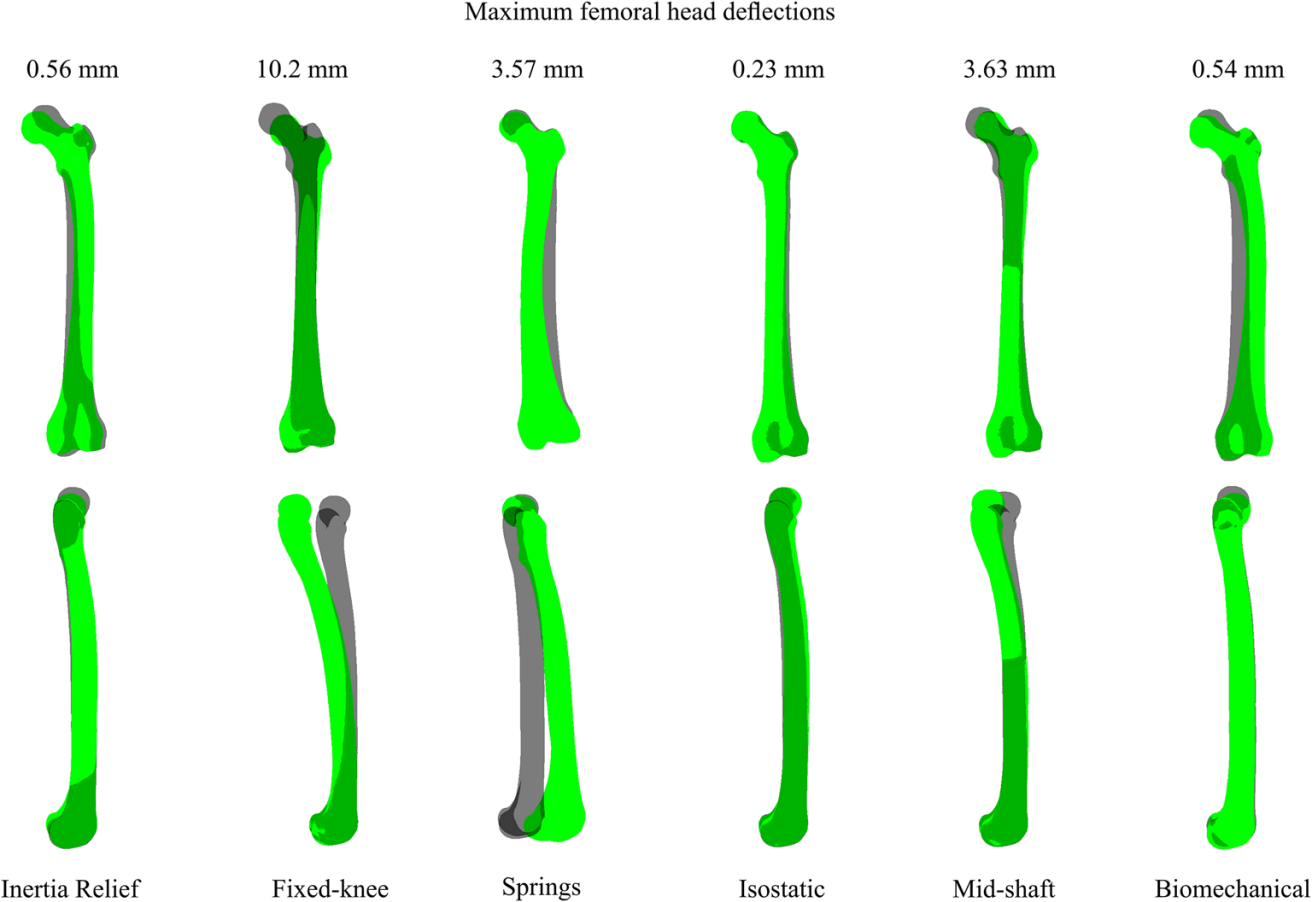
Deflected femur (green) in posterior (top row) and lateral (bottom row) views overlaid on neutral (undeformed) femur geometry (grey) during midstance. All deflections are uniformly scaled tenfold for clarity.

**Figure 5:**
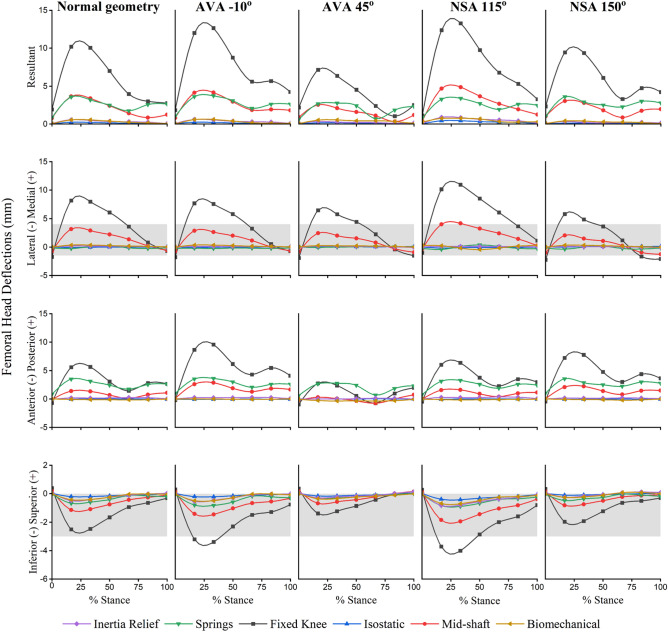
Femoral head deflections across the stance phase for the following configurations: normal (neutral undeformed) femur geometry (column 1), extreme versions (columns 2 and 3), and extreme neck-shaft angles (columns 4 and 5). Grey bands (present in rows 2 and 4) represent experimental measurements of the FHD reported by Taylor et al.^41^. AVA -10°—anteversion angle of -10°; AVA 45°—anterversion angle of 45°; NSA 115°—neck-shaft angle of 115°; NSA 150°—neck-shaft angle of 150°.

For all normal and pathological femur geometries, the *fixed knee* method resulted in the highest FHD in all anatomical directions with a maximum resultant FHD of 10.2 mm during midstance (Figs. 4, 5). Similarly, the *fixed knee* method produced the highest FHD in models with femoral deformity, with a resultant FHD of 13.28 mm in the case of NSA 115°. For all models, *springs*, *isostatic*, *IR*, and *biomechanical* methods were within 5% of each other in terms of the FHD in the medial–lateral direction. The *biomechanical* method showed the highest agreement (R^2^ = 0.96, nRMSE = 2.21) with the *IR* method in the superior–inferior FHD compared to all other constraint sets during stance for all femur geometries. A detailed statistical comparison of FHD values across various boundary conditions compared to the benchmark *IR* method is presented in Supplementary Material, Table S3.

### Peak von Mises stress

**Figure 6:**
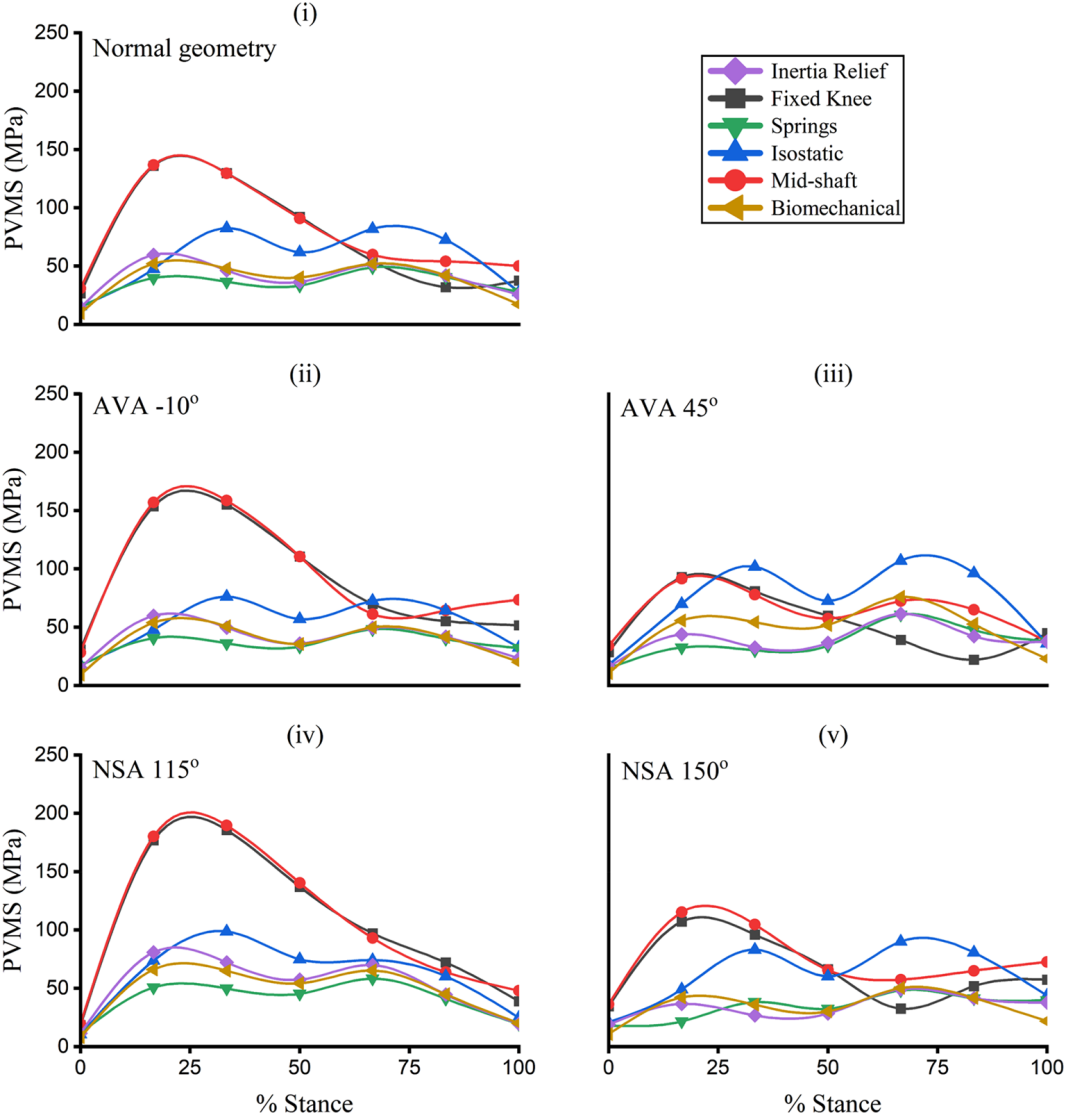
The Peak von Mises stress (PVMS) developed in femurs across the stance phase of gait. (**i**) Normal (undeformed) geometry, (**ii**)  -10° anteversion geometry, (**iii**) 45° anteversion model, (**iv**) 115° neck-shaft angle model, and (**v**) 150° neck-shaft angle model. AVA -10°—anteversion angle of -10°; AVA 45°—anteversion angle of 45°; NSA 115°—neck-shaft angle of 115°; NSA 150°—neck-shaft angle of 150°.

The PVMS simulated in the femur was substantially influenced by the boundary conditions (Fig. 6). Apart from the AVA 45° geometry, *fixed knee* and *mid-shaft* methods resulted in the highest PVMS for all femur geometries. The *springs* method resulted in the lowest PVMS across all femur geometries. Compared to the benchmark *IR* study, the *biomechanical* method showed the highest agreement (R^2^ > 0.92) for normal, AVA -10°, and NSA 115° femur geometries. In NSA 150°, the *biomechanical* model showed the best agreement with the *IR* method (R^2^ = 0.61, nRMSE = 0.23). However, in the AVA 45°, the *springs* method showed the highest agreement (R^2^ = 0.89, nRMSE = 0.13) with the *IR* method followed by the *biomechanical* method (R^2^ = 0.71, nRMSE = 0.37). A detailed statistical comparison of PVMS values across the various boundary conditions compared to the *IR* method is presented in Supplementary Material, Table S4.

### Cortical strain

**Figure 7:**
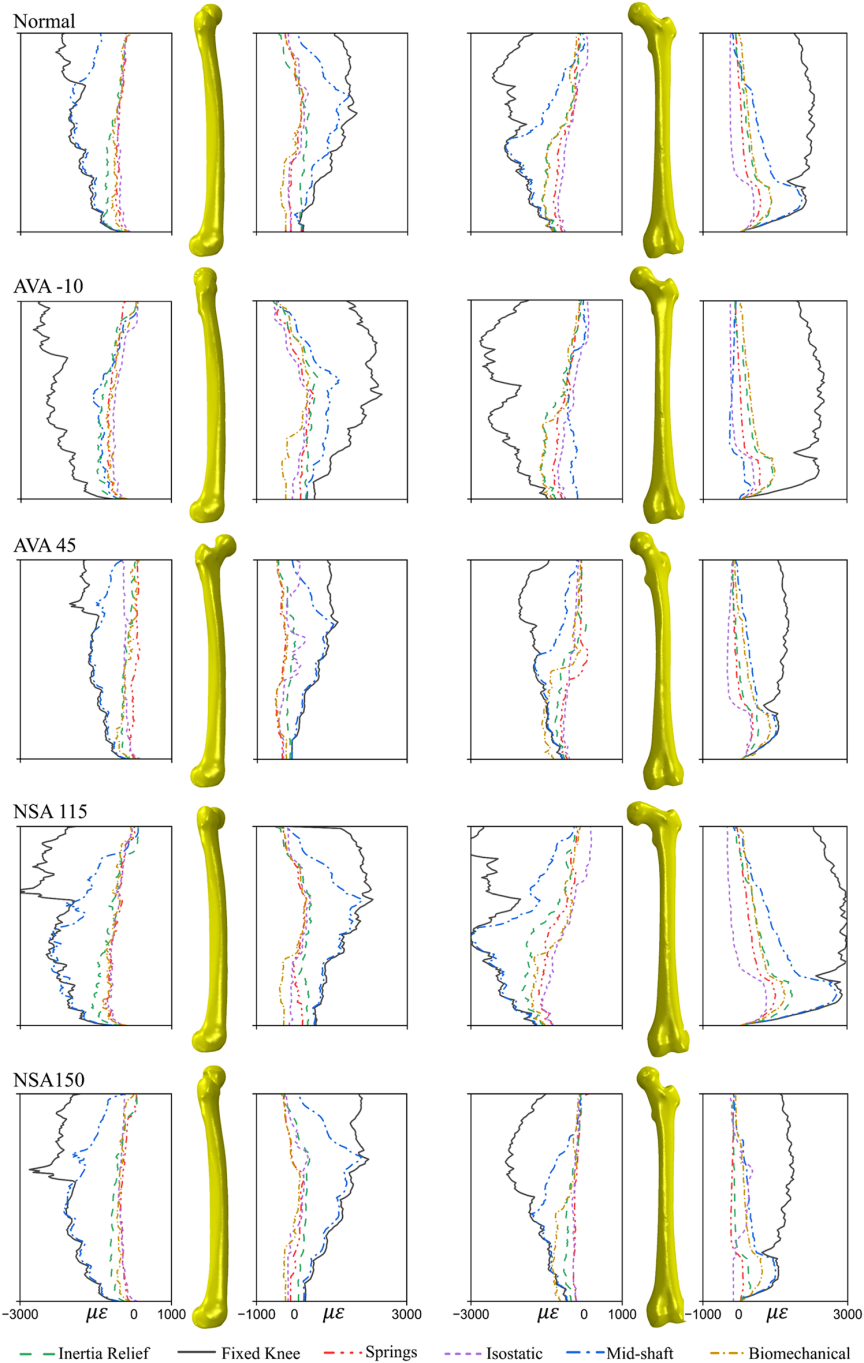
The absolute principal cortical microstrains measured along predefined paths on the medial (third column), lateral (fourth column), posterior (first column), and anterior (second column) femoral cortex for the normal, extreme anteversion, and extreme neck-shaft angle geometries. AVA -10°—anteversion angle of -10°; AVA 45°—anteversion angle of 45°; NSA 115°—neck-shaft angle of 115°; NSA 150°—neck-shaft angle of 150°.

The *fixed knee* method resulted in the highest cortical strains across the femoral diaphysis (Fig. 7). In the NSA 115° femur, posterior and medial cortical surfaces experienced in excess of 3000 µε in compression, while the lateral cortex experiences approximately 3000 µε in tension (Fig. 7). The *isostatic* method produced the lowest cortical strains for all femurs, with the exception of AVA -10°. In the normal femur geometry, *isostatic*, *IR*, and *biomechanical* methods resulted in similar cortical strains of < 1100 µε. Strain patterns across all cortical aspects were similar between *IR* and *biomechanical* methods for the normal femur geometry, and similar in medial and lateral cortex surfaces for all femur geometries (Fig. 7).

## Discussion

The primary finding of this study is that commonly implemented approaches to constraining the isolated femur resulted in non-physiological bone mechanics (e.g., critical stresses during the non-injurious task of walking) or supra-physiological strains. The *biomechanical* method performed similar to the benchmark *IR* method and can therefore be used in conditions where the *IR* method is not supported (i.e., multi-part models in Abaqus, such as a femur combined with implants). Although the *biomechanical* method requires development of adequate constraints and subsequent validation for other isolated long bones, this study confirms the superior performance of the *biomechanical* method for estimating stresses and strains in the isolated femur compared to other methods reported in the literature.

### Performance of biomechanical compared to the IR method

The *biomechanical* method developed for this paper performed similar to the *IR* method in terms of simulated FHD, PVMS, and cortical strains, and did not impose artificial constraints on femoral head movement. The *biomechanical* method thus replicated natural femoral head motion within the acetabulum across the full walking gait cycle. Results indicate the *biomechanical* method is an appropriate boundary condition for studies focused on FHD in the isolated femur.

In terms of the simulated PVMS, the *biomechanical* method performed similar to the *IR* method. This indicates that the *biomechanical* method is an appropriate method for establishing boundary conditions for subsequent stress analysis of isolated femurs. Moreover, the *biomechanical* method induced cortical strains in the femur diaphysis closely mimicking strains generated by the benchmark *IR* method, thus indicating its simulations are physiological. Within the Abaqus computational framework, the *biomechanical* method offers an advantage over the *IR* method as it can be used with multi-component models, such as bone fractures and implants, whereas the *IR* currently method cannot. Consequently, the *biomechanical* method may be most appropriate when the *IR* method is not supported due to software or model considerations.

### Performance of commonly used boundary conditions against inertia relief method

Consistent with results from Speirs et al.^35^, the commonly used *fixed knee* method produced non-physiological femur biomechanics for both normal and pathologic female geometries. The femoral head is naturally constrained by the acetabulum, thereby preventing an excessive FHD. However, the *fixed knee* method does not account for this proximal constraint of the femur resulting in overestimation of the FHD. As with the *fixed knee* method, a higher FHD compared to the benchmark in the *mid-shaft* method highlights the importance of including a constraint for the proximal femur when analyzing isolated femurs. Compared to the *IR* method, the *springs* method simulated a larger FHD due to the number and stiffness of the springs used to constrain the femur head. In contrast, the *isostatic* method results in a negligible FHD, especially in the anterior–posterior and medial–lateral directions due to the artificial constraints imposed on the femur head such that the effects of the applied anterior–posterior and medial–lateral components of the hip joint contact force are eliminated. This approach may be inadequate for simulations where medial–lateral and/or anterior–posterior forces are large, which would include most cases of human locomotion as they involve distinct phases of breaking and propulsion aligned primarily with the anterior–posterior direction of the femur (and other lower limb bones).

The *fixed knee* and *mid-shaft* methods clearly lead to overestimation of the PVMS, which is relevant when the PVMS value is used to assess design of implants, stress-shielding, and implant performance. Conversely, for the *springs* and *isostatic* methods, PVMS values were within ranges reported in literature for normal femur geometry^79^. However, the *springs* method was found to be highly sensitive to the mechanical properties and attachment points of the *springs*, which may weaken its potential due to reliability and reproducibility. For this study, the number and stiffness of the springs were tuned to avoid stress concentrations at regions where springs attached to bone.

Our findings show that, across all boundary conditions and for the full walking gait cycle, the lateral femur cortex is subjected to tensile strains, while the medial cortex is subject to compressive strains, which is consistent with results from Taylor et al.^41^. The high magnitudes of cortical strains in the *fixed knee* and *mid-shaft* methods can be explained by the excessive FHD induced by these boundary conditions. The findings of this study highlight that commonly used boundary conditions in literature lacks the ability to produce physiological mechanics in isolated femurs.

### Pathological femur geometries

The introduction of femoral pathologies, specifically atypical neck shaft and version angles, influenced the effects of boundary conditions on isolated femur mechanics. The FHD predicted by the *fixed knee* method exceeded physiological limits in the medial–lateral direction for all pathological femur geometries. The *fixed knee* method also produced a non-physiological FHD in the superior–inferior direction for AVA -10° and NSA 115°. Similarly, the *mid-shaft* method resulted in a non-physiological FHD in medial–lateral directions in the NSA 115° model and values near physiological limit for the other pathological femur geometries. For all tested pathological femur geometries, the *springs* method resulted in a physiological FHD in superior-inferior and medial–lateral directions, but in anterior–posterior direction it performed similarly to the *fixed knee* and *mid-shaft* methods. Our preliminary in-house analyses (not reported here) found that the FHD was highly sensitive to spring stiffness, attachment points, and number of spring elements. Therefore, the *springs* method should be employed with caution in the FEA of isolated femurs, as implementations will vary across laboratories making comparison challenging and potentially of little value. In contrast to the above methods, the *isostatic* method simulated a relatively modest FHD, with resultant deflections < 0.25 mm. These small FHD values may be a result of the *isostatic* method eliminating medial–lateral and anterior–posterior components of the hip contact force by constraining the femoral head. Importantly, both the *IR* and *biomechanical* methods resulted in physiological FHD simulations, and similar trends in FHD values were observed throughout all pathological femur geometries for both methods.

The *fixed knee* and *mid-shaft* methods resulted in simulated PVMS values more than yield stress for healthy bone in several femur geometries, including anteverted, retroverted, *coxa vara*, and *coxa valga*^79^. Similarly, the *isostatic* method simulated a PVMS more than bone yield stress in severely anteverted femurs, indicating this approach overestimated stress in pathological geometries. In contrast, *IR*, *biomechanical*, and *springs* methods predicted sub-critical PVMS results in bone across all femur geometries. The *biomechanical* method performed similar to the *IR* method in terms of simulated PVMS values for pathological femurs, indicating that it is an appropriate method for constraining abnormal femur geometries.

The *fixed knee* method resulted in the highest simulated cortical strains, especially in the proximal area of the femur with cortical strains exceeding 3000 µε in *coxa vara* femur geometry. The *mid-shaft* method also produced high magnitudes of cortical strains similar to the *fixed knee* method in anteverted, *coxa valga*, and *coxa vara* femur geometries. In contrast, the *isostatic*, *springs*, *IR*, and *biomechanical* methods show similar cortical strain patterns across pathological femur geometries. Among these methods, the *biomechanical* method again showed the highest similarity to the *IR* method in pathological femurs indicating that it would be an appropriate method for study of cortical strains in an isolated femur.

### Strengths, limitations, and future directions

This study is the first to run a comprehensive benchmarking analysis of the effects of boundary conditions on isolated femur mechanics for both normal and pathological femurs across the stance phase of the walking gait cycle. This research demonstrates strong predictive capabilities, employing a sequentially linked NMSK-FEA workflow to accurately determine loading conditions, assessing multiple relevant FEA outcome measures, and comparing widely used boundary conditions to the benchmarking *IR* method. The presented workflow can be adopted and extended to other long bones and FEA models involving complex loading inputs to simulate physiological bone mechanics.

This study highlights the pivotal role of boundary condition selection in determining the accuracy of FEA outcomes within clinical contexts. Arbitrary, or even popular, approaches to establishing boundary conditions may result in spurious simulated mechanics, thus undermining the integrity of the FEA process to inform clinical decision-making. Our analysis of deformed femurs provides a key observation: a constraining method that performs well for intact typical femurs might not well simulate physiological conditions for pathological femurs. This indicates the necessity for careful selection of boundary conditions, tailored to the specific research inquiry or the goals of the analysis. Further, the *biomechanical* boundary condition presented here provides a method for physiological constraint that can be applied to multi-component models, including those with implants and dissections, which is critical for many clinical applications (i.e., surgical planning and evaluation) where implants and surgical corrections are involved. Although further research is warranted to directly compare these methods in the context of implanted femurs, preliminary evaluations suggest a pronounced sensitivity to the constraining method within surgically modified models, emphasizing the need for a more reliable boundary condition as developed in this study. This is of great relevance to orthopedic and rehabilitation practices, influencing various clinical applications such as surgical planning, implant design, predictive modeling, and in-silico trials. The insights derived from this research have the potential to significantly inform and improve decision-making processes in these vital areas of medical practice.

Although this study generates important insights, it has limitations that should be acknowledged. First, muscle and joint contact forces used in the boundary conditions were from NMSK simulations of walking gait. Future studies should incorporate muscle and joint contact forces from other motor tasks to assess if the findings from walking gait can be generalized to other motor tasks or if boundary condition effects on isolated femur mechanics are highly task specific. Second, the significant function of the cartilage layers on both hip and knee joints was neglected in NMSK and FEA processes^80,81^. Lower limb cartilages play a crucial role at the articulations between body segments (e.g., hip and knee joints) as they provide an ultra-low friction lubricated bearing surface and aid the distribution of joint contact forces across the surfaces of the joint and to the underlying skeleton. This mechanical function should be modelled in future studies to determine its significance in FEA of lower limb bones when compared to a distributed load approach over representative surface areas as employed in this study. Third, the femur pathologies studied here were defined as clinically extreme. Less extreme deformations of the femur, as well as complex combinations of deformities, should be assessed in future studies as these indications are also treated clinically. Furthermore, identical muscle and joint contact forces were applied across all healthy and pathological models for all boundary conditions. Although the MSK model could have been adapted to simulate the pathologies and generate muscle and joint contact forces, the lack of pathological gait and EMG data precludes the model’s validation. Future studies should aim to evaluate these boundary conditions using actual pathological geometries, gait data, and EMG data. Fourth, the femur geometry in this study was meshed using tetrahedral elements. Studies have shown that the mesh type can substantially affect mechanics simulated through FEA. We performed mesh convergence analysis to ensure our results were not influenced by mesh size. However, other element types could be considered and investigated in future studies, especially if bending stresses near the surface were to be reported. Fifth, the analysis neglected the effects of acceleration and gravitational forces within the femur body generated due to the dynamics of the thigh segment and its location within a gravitational field, except for the case of the *IR* method, which intrinsically incorporates these effects. These forces were comparatively lower than muscle and joint contact forces (by an order of magnitude) and were assumed to have insignificant effects on the simulate bone mechanics due to the analysis of walking gait. This assessment would need to be re-evaluated for gaits involving larger accelerations and dynamic forces.

## Conclusions

We demonstrated that femoral head deflection, peak von Mises stresses, and cortical strains are all highly sensitive to boundary conditions. The newly developed *biomechanical* method generates physiological simulations of isolated bone mechanics and is applicable to a wide range of femur models, including deformed, implanted, and fractured models in any FEA software package. Based on our findings, we recommend the *biomechanical* method as the preferred approach for constraining the femur in FEA, although the choice of boundary conditions should be carefully considered based on the specific research question, model requirements, and desired outcomes.

### Supplementary Information

Supplementary Information.

## Data Availability

The datasets generated during and/or analysed during the current study are available from the corresponding author on reasonable request.

## References

[1] Kaupp SM, Mann KA, Miller MA, Damron TA (2021). Predicting fracture risk in patients with metastatic bone disease of the femur: A pictorial review using three different techniques. Adv. Orthop..

[2] Longo UG, Koh J (2021). FEA applications for orthopedics: An overview. Orthopaedic Biomechanics in Sports Medicine.

[3] Meslier QA, Shefelbine SJ (2023). Using finite element modeling in bone mechanoadaptation. Curr. Osteoporos. Rep..

[4] MacLeod AR (2021). Personalised high tibial osteotomy has mechanical safety equivalent to generic device in a case–control in silico clinical trial. Commun. Med..

[5] Duda GN (1998). Influence of muscle forces on femoral strain distribution. J. Biomech..

[6] Armillotta N, Bori E, Innocenti B (2023). Finite element analysis of malposition in bi-unicompartmental knee arthroplasty. Arch. Orthop. Trauma Surg..

[7] Lu M (2021). “Fishing net” suture augmenting tension-band wiring fixation in the treatment of inferior pole fracture of the patella. Arch. Orthop. Trauma Surg..

[8] Burastero G (2020). Use of porous custom-made cones for meta-diaphyseal bone defects reconstruction in knee revision surgery: A clinical and biomechanical analysis. Arch. Orthop. Trauma Surg..

[9] Shash YH, El-Wakad MT, El-Dosoky MAA, Dohiem MM (2023). Evaluation of stresses on mandible bone and prosthetic parts in fixed prosthesis by utilizing CFR-PEEK, PEKK and PEEK frameworks. Sci. Rep..

[10] Toyohara R (2020). Finite element analysis of load transition on sacroiliac joint during bipedal walking. Sci. Rep..

[11] Deng C, Gillette JC, Derrick TR (2021). Finite element analysis of femoral neck strains during stair ascent and descent. Sci. Rep..

[12] Heller MO (2011). Influence of prosthesis design and implantation technique on implant stresses after cementless revision THR. J. Orthop. Surg. Res..

[13] Robinson DL (2020). Load response of an osseointegrated implant used in the treatment of unilateral transfemoral amputation: An early implant loosening case study. Clin. Biomech..

[14] Huang D, Hu Z, Feng W, Wang X (2023). Dual plate has better biomechanical stability than hook plate or superior single plate for the fixation of unstable distal clavicle fractures: A finite element analysis. Arch. Orthop. Trauma Surg..

[15] Bockmann B (2023). Bone tunnel placement influences shear stresses at the coracoid process after coracoclavicular ligament reconstruction: A finite element study and radiological analysis. Arch. Orthop. Trauma Surg..

[16] Fletcher JW (2019). The influence of screw length on predicted cut-out failures for proximal humeral fracture fixations predicted by finite element simulations. Arch. Orthop. Trauma Surg..

[17] Jang YW (2018). Role of an anatomically contoured plate and metal block for balanced stability between the implant and lateral hinge in open-wedge high-tibial osteotomy. Arch. Orthop. Trauma Surg..

[18] Lee JS, Kim KG, Yoon YC (2023). Biomechanical performance evaluation of a modified proximal humerus locking plate for distal humerus shaft fracture using finite element analysis. Sci. Rep..

[19] Tauviqirrahman M (2023). Analysis of contact pressure in a 3D model of dual-mobility hip joint prosthesis under a gait cycle. Sci. Rep..

[20] Moideen ISM, Lim CT, Yeow RCH, Chong DYR (2020). Finite element analysis of bone-prosthesis interface micromotion for cementless talar component fixation through critical loading conditions. Int. J. Numer. Methods Biomed. Eng..

[21] Samsami S, Saberi S, Sadighi S, Rouhi G (2015). Comparison of three fixation methods for femoral neck fracture in young adults: Experimental and numerical investigations. J. Med. Biol. Eng..

[22] Kang K-T, Son J, Baek C, Kwon O-R, Koh Y-G (2018). Femoral component alignment in unicompartmental knee arthroplasty leads to biomechanical change in contact stress and collateral ligament force in knee joint. Arch. Orthop. Trauma Surg..

[23] El-Zayat BF (2016). Fixation techniques and stem dimensions in hinged total knee arthroplasty: A finite element study. Arch. Orthop. Trauma Surg..

[24] Gubaua JE, Dicati GWO, Mercuri EGF, Pereira JT (2020). Simulation of bone remodeling around a femoral prosthesis using a model that accounts for biological and mechanical interactions. Med. Eng. Phys..

[25] Huiskes R, Weinans H, Van Rietbergen B (1992). The relationship between stress shielding and bone resorption around total hip stems and the effects of flexible materials. Clin. Orthop. Relat. Res..

[26] Hildebrand M (2022). Development and characterization of a predictive microCT-based non-union model in Fischer F344 rats. Arch. Orthop. Trauma Surg..

[27] Su K, Yuan L, Yang J, Du J (2019). Numerical simulation of mandible bone remodeling under tooth loading: A parametric study. Sci. Rep..

[28] Cho BW (2021). Anatomical tibial component is related to more medial tibial stress shielding after total knee arthroplasty in Korean patients. Knee Surg. Sports Traumatol. Arthrosc..

[29] Filardi V (2018). Flatfoot and normal foot a comparative analysis of the stress shielding. J. Orthop..

[30] Luo C (2020). Femoral stress changes after total hip arthroplasty with the ribbed prosthesis: A finite element analysis. BioMed. Res. Int..

[31] Prochor P, Sajewicz E (2019). The influence of geometry of implants for direct skeletal attachment of limb prosthesis on rehabilitation program and stress-shielding intensity. BioMed. Res. Int..

[32] Heyland M, Checa S, Kendoff D, Duda GN (2019). Anatomic grooved stem mitigates strain shielding compared to established total hip arthroplasty stem designs in finite-element models. Sci. Rep..

[33] Bayoglu R, Okyar AF (2015). Implementation of boundary conditions in modeling the femur is critical for the evaluation of distal intramedullary nailing. Med. Eng. Phys..

[34] Heyland M (2015). Selecting boundary conditions in physiological strain analysis of the femur: Balanced loads, inertia relief method and follower load. Med. Eng. Phys..

[35] Speirs AD, Heller MO, Duda GN, Taylor WR (2007). Physiologically based boundary conditions in finite element modelling. J. Biomech..

[36] Wijker J (2004). Free-free Dynamic Systems, Inertia Relief.

[37] Anvari, M. & Beigi, B. Automotive body fatigue analysis–inertia relief or transient dynamics? *SAE Technical Paper* (1999)

[38] Bryer G, Eccles C (2011). Structural optimization for vehicle dynamics loadcases. SAE Int. J. Passeng. Cars Electron. Electr. Syst..

[39] Liao, L. *52nd AIAA/ASME/ASCE/AHS/ASC Structures, Structural Dynamics and Materials Conference.*10.2514/6.2011-2002 (2002).

[40] Dassault Systèmes Simulia Corp. (2022).

[41] Taylor M, Tanner K, Freeman M, Yettram A (1996). Stress and strain distribution within the intact femur: Compression or bending?. Med. Eng. Phys..

[42] Voo L, Armand M, Kleinberger M (2004). Stress fracture risk analysis of the human femur based on computational biomechanics. Johns Hopkins Appl. Tech. Dig..

[43] Jung IJ, Kim JW (2021). Differences in femur geometry and bone markers in atypical femur fractures and the general population. Sci. Rep..

[44] Lafortune M, Cavanagh P, Sommer Iii H, Kalenak A (1992). Three-dimensional kinematics of the human knee during walking. J. Biomech..

[45] Wei YC (2022). Finite element analysis on femur subjected to knee joint forces during incline-decline walking. Int. J. Adv. Technol. Eng. Explor..

[46] Nithin Kumar KC, Griya N, Shaikh A, Chaudhry V, Chavadaki S (2019). Structural analysis of femur bone to predict the suitable alternative material. Mater. Today Proc..

[47] Nolte D, Bull AMJ (2019). Femur finite element model instantiation from partial anatomies using statistical shape and appearance models. Med. Eng. Phys..

[48] Phillips ATM (2009). The femur as a musculo-skeletal construct: A free boundary condition modelling approach. Med. Eng. Phys..

[49] Reina-Romo E, Rodriguez-Valles J, Sanz-Herrera JA (2018). In silico dynamic characterization of the femur: Physiological versus mechanical boundary conditions. Med. Eng. Phys..

[50] Geraldes DM, Phillips AT (2014). A comparative study of orthotropic and isotropic bone adaptation in the femur. Int. J. Numer. Methods Biomed. Eng..

[51] Phillips ATM, Villette CC, Modenese L (2015). Femoral bone mesoscale structural architecture prediction using musculoskeletal and finite element modelling. Int. Biomech..

[52] Harding L (2003). Posterior-anterior glide of the femoral head in the acetabulum: A cadaver study. J. Orthop. Sports Phys. Ther..

[53] Kainz H (2017). Reliability of four models for clinical gait analysis. Gait Posture.

[54] Hermens HJ, Freriks B, Disselhorst-Klug C, Rau G (2000). Development of recommendations for SEMG sensors and sensor placement procedures. J. Electromyogr. Kinesiol..

[55] Mantoan A (2015). MOtoNMS: A MATLAB toolbox to process motion data for neuromusculoskeletal modeling and simulation. Source Code Biol. Med..

[56] Delp SL (2007). OpenSim: Open-source software to create and analyze dynamic simulations of movement. IEEE Trans. Biomed. Eng..

[57] Hewett TE, Myer GD, Roewer BD, Ford KR (2012). Letter to the editor regarding “Effect of low pass filtering on joint moments from inverse dynamics: Implications for injury prevention”. J. Biomech..

[58] Winter DA (2009). Biomechanics and Motor Control of Human Movement.

[59] Rajagopal A (2016). Full-body musculoskeletal model for muscle-driven simulation of human gait. IEEE Trans. Biomed. Eng..

[60] Modenese L, Ceseracciu E, Reggiani M, Lloyd DG (2016). Estimation of musculotendon parameters for scaled and subject specific musculoskeletal models using an optimization technique. J. Biomech..

[61] Handsfield GG, Meyer CH, Hart JM, Abel MF, Blemker SS (2014). Relationships of 35 lower limb muscles to height and body mass quantified using MRI. J. Biomech..

[62] van Arkel RJ, Modenese L, Phillips AT, Jeffers JR (2013). Hip abduction can prevent posterior edge loading of hip replacements. J. Orthop. Res..

[63] Pizzolato C (2015). CEINMS: A toolbox to investigate the influence of different neural control solutions on the prediction of muscle excitation and joint moments during dynamic motor tasks. J. Biomech..

[64] Sartori M, Reggiani M, Farina D, Lloyd DG (2012). EMG-driven forward-dynamic estimation of muscle force and joint moment about multiple degrees of freedom in the human lower extremity. PLoS ONE.

[65] Sartori M, Farina D, Lloyd DG (2014). Hybrid neuromusculoskeletal modeling to best track joint moments using a balance between muscle excitations derived from electromyograms and optimization. J. Biomech..

[66] Meinders E (2022). Activation of the deep hip muscles can change the direction of loading at the hip. J. Biomech..

[67] Savage TN (2022). Hip contact force magnitude and regional loading patterns are altered in those with femoroacetabular impingement syndrome. Med. Sci. Sports Exerc..

[68] Hoang HX, Diamond LE, Lloyd DG, Pizzolato C (2019). A calibrated EMG-informed neuromusculoskeletal model can appropriately account for muscle co-contraction in the estimation of hip joint contact forces in people with hip osteoarthritis. J. Biomech..

[69] Starkey SC (2022). Effect of functional weightbearing versus non-weightbearing quadriceps strengthening exercise on contact force in varus-malaligned medial knee osteoarthritis: A secondary analysis of a randomized controlled trial. The Knee.

[70] Starkey SC (2020). Effect of exercise on knee joint contact forces in people following medial partial meniscectomy: A secondary analysis of a randomised controlled trial. Gait Posture.

[71] Chen D (2017). Three-dimensional reconstructions in spine and screw trajectory simulation on 3D digital images: A step by step approach by using Mimics software. J. Spine Surg..

[72] Schileo E (2008). An accurate estimation of bone density improves the accuracy of subject-specific finite element models. J. Biomech..

[73] Taddei F, Cristofolini L, Martelli S, Gill H, Viceconti M (2006). Subject-specific finite element models of long bones: An in vitro evaluation of the overall accuracy. J. Biomech..

[74] Li X (2015). Developing CT based computational models of pediatric femurs. J. Biomech..

[75] Muller M (2008). Prediction of bone mechanical properties using QUS and pQCT: Study of the human distal radius. Med. Eng. Phys..

[76] Ejnisman L (2013). Relationship between femoral anteversion and findings in hips with femoroacetabular impingement. Orthopedics.

[77] O’Sullivan IR, Schégl ÁT, Varga P, Than P, Vermes C (2021). Femoral neck-shaft angle and bone age in 4- to 24-year-olds based on 1005 EOS three-dimensional reconstructions. J. Pediatr. Orthop. B.

[78] Oladeji AK (2023). Blade plate versus locking plate fixation of proximal femoral varus osteotomy in children with cerebral palsy. J. Pediatr. Orthop. B.

[79] Öhman C (2011). Compressive behaviour of child and adult cortical bone. Bone.

[80] Adouni MA-O, Alkhatib F, Gouissem A, Faisal TR (2023). Knee joint biomechanics and cartilage damage prediction during landing: A hybrid MD-FE-musculoskeletal modeling. PLoS ONE.

[81] Al Khatib F, Gouissem A, Mbarki R, Adouni M (2022). Biomechanical characteristics of the knee joint during gait in obese versus normal subjects. Int. J. Environ. Res. Public Health.

