# Supplementary material for: Effect of different constraining boundary conditions on simulated femoral stresses and strains during gait
Source: Sci Rep. 2024 May 11;14:10808. doi: 10.1038/s41598-024-61305-x (PMC11088641; doi:10.1038/s41598-024-61305-x)
Supplement: Supplementary file 1 — Supplementary Information. [file 41598_2024_61305_MOESM1_ESM.docx]

**Supplementary material**

# Systematic Review

We surveyed the different approaches to specifying, or creating, the boundary conditions used when predicting the stress state of an isolated femur. The survey comprised of a literature search with Scopus, Web of Science, and PubMed for studies published between 1987 and 31/05/2023. The following keywords were used: “finite element” OR “numerical” OR “*in silico*” AND “boundary condition*” OR “constraint*” AND “femur” to search within the abstract, keywords, and the title of the documents. A total of 719 results were returned with 294 duplicates. The 425 unique results were screened for titles and abstracts by two of the co-authors (EEA and AYB), and 85 full-text studies were assessed for eligibility.

Inclusion criteria were as follows: full text available in English, study specimen is a human femur, and finite element model is a full-length femur. Exclusion criteria were as follows: study specimen is not a human femur, studies that are not published in English, the test specimen is a proximal or distal half-femur, and the study is a conference paper. Forty-two articles met the inclusion criteria and were included in the rapid review. The PRISMA diagram and study details are presented in Table S1 and Fig. S1.

**Table S1**. Summary of the review showing the loads (muscle forces and joint contact forces) and boundary conditions applied to the finite element studies. *PF = patellofemoral

| **Boundary condition** | **Study** | **Muscle forces** | | | **Joint contact forces** | | |
| --- | --- | --- | --- | --- | --- | --- | --- |
|  |  | None | Simplified | Complex | Hip | Knee | PF |
| Inertia relief | Heyland, et al. [1] |  | ✓ |  | ✓ |  |  |
| Fixed knee | Ferré, et al. [2] |  | ✓ |  | ✓ |  |  |
|  | Speirs, et al. [3] |  |  | ✓ | ✓ | ✓ | ✓ |
|  | Gang, et al. [4] | ✓ |  |  | ✓ |  |  |
|  | Bayoglu and Okyar [5] | ✓ |  |  | ✓ |  |  |
|  | Gok and Inal [6] | ✓ |  |  | ✓ |  |  |
|  | Heyland, et al. [1] |  | ✓ |  | ✓ |  |  |
|  | Zhou, et al. [7] | ✓ |  |  | ✓ |  |  |
|  | Celik, et al. [8] |  | ✓ |  | ✓ |  |  |
|  | Gok, et al. [9] | ✓ |  |  | ✓ |  |  |
|  | Abdullah, et al. [10] | ✓ |  |  | ✓ |  |  |
|  | Gok, et al. [11] | ✓ |  |  | ✓ |  |  |
|  | Chen and Mao [12] | ✓ |  |  | ✓ |  |  |
|  | Chethan, et al. [13] | ✓ |  |  | ✓ |  |  |
|  | Inal, et al. [14] | ✓ |  |  |  |  |  |
|  | Gok, et al. [15] | ✓ |  |  | ✓ |  |  |
|  | Kluess, et al. [16] | ✓ |  |  | ✓ |  |  |
|  | Gok, et al. [17] | ✓ |  |  | ✓ |  |  |
|  | Rose [18] | ✓ |  |  | ✓ |  |  |
|  | Rathor, et al. [19] |  | ✓ |  | ✓ |  |  |
|  | Kumar, et al. [20] | ✓ |  |  | ✓ |  |  |
|  | Altai, et al. [21] |  |  | ✓ | ✓ | ✓ |  |
|  | Gee, et al. [22] | ✓ |  |  | ✓ |  |  |
|  | Joshi, et al. [23] |  | ✓ |  | ✓ |  |  |
|  | Fritz, et al. [24] |  | ✓ |  | ✓ |  |  |
|  | Heller [25] |  |  | ✓ | ✓ | ✓ |  |
| Springs | Phillips [26] |  |  | ✓ | ✓ | ✓ | ✓ |
|  | Frydrysek, et al. [27] | ✓ |  |  | ✓ |  |  |
| Isostatic | Speirs, et al. [3] |  |  | ✓ | ✓ | ✓ | ✓ |
|  | Andreaus and Colloca [28] |  | ✓ |  | ✓ |  |  |
|  | Behrens, et al. [29] |  | ✓ |  | ✓ |  |  |
|  | Lerch, et al. [30] |  | ✓ |  | ✓ |  |  |
|  | Bah, et al. [31] |  | ✓ |  | ✓ |  |  |
|  | Bayoglu and Okyar [5] |  | ✓ |  | ✓ |  |  |
|  | Heyland, et al. [1] |  | ✓ |  | ✓ |  |  |
|  | Haider, et al. [32] |  |  | ✓ | ✓ |  |  |
|  | Reina-Romo, et al. [33] | ✓ |  |  |  |  |  |
|  | Heyland, et al. [34] |  | ✓ |  | ✓ |  |  |
|  | Altai, et al. [21] |  |  | ✓ | ✓ | ✓ |  |
|  | Jitprapaikulsarn, et al. [35] |  | ✓ |  | ✓ |  |  |
|  | Heller [25] |  |  | ✓ | ✓ | ✓ |  |
|  | Jitprapaikulsarn, et al. [36] |  | ✓ |  | ✓ |  |  |
| Mid-shaft | Speirs, et al. [3] |  |  | ✓ | ✓ | ✓ | ✓ |
|  | Szwedowski, et al. [37] |  |  | ✓ | ✓ |  |  |
|  | Abdullah, et al. [10] | ✓ |  |  | ✓ |  |  |
|  | Reina-Romo, et al. [33] | ✓ |  |  |  |  |  |
| Other | Phillips [26] |  |  | ✓ | ✓ | ✓ | ✓ |
|  | Gok, et al. [9] | ✓ |  |  |  |  |  |
|  | Reina-Romo, et al. [33] | ✓ |  |  |  |  |  |
|  | Nithin Kumar, et al. [38] | ✓ |  |  |  | ✓ |  |
|  | Nolte and Bull [39] | ✓ |  |  |  | ✓ | ✓ |
|  | Wei, et al. [40] | ✓ |  |  |  | ✓ |  |
|  | Oza, et al. [41] | ✓ |  |  | ✓ |  |  |
|  | Inacio, et al. [42] | ✓ |  |  | ✓ |  |  |

**Fig. S1**. Systematic search strategy results.

# Neuromusculoskeletal modelling

Table S2 shows the mapping of 12 muscle excitations across 23 MTUs, which were utilized for the subsequent execution of CEINMS in EMG-assisted mode.

| **Experimental EMG** | **MTU distributions** |
| --- | --- |
| Biceps femoris long head | bflh_r, bfsh_r, addbrev_r, addlong_r, addmagDist_r, addmagIsch_r, addmagMid_r, addmagProx_r |
| Gastrocnemius lateralis | gaslat_r, gasmed_r, |
| Gluteus maximus | glmax1_r, glmax2_r, glmax3_r, piri_r, glmin1_r, glmin2_r, glmin_3 |
| Gluteus medius | glmed1_r, glmed2_r, glmed3_r, glmin1_r, glmin2_r, glmin_3, tibpost_r, piri_r |
| Gracilis | grac_r |
| Rectus femoris | recfem_r, iliacus_r, perbrev_r, perlong_r, psoas_r |
| Sartorius | sart_r, ehl_r |
| Semitendinosus | semiten_r, semimem_r, edl_r |
| Tensor fasciae latae | tfl_r, soleus_r |
| Tibialis anterior | tibant_r, fdl_r |
| Vastus lateralis | vaslat_r, vasint_r |
| Vastus medialis | vasmed_r, vasint_r |

Table S2. Experimental muscle excitations distributed to 23 MTUs. The MTU label convention were adopted from the OpenSim model.

## Mesh convergence analysis

Fig. S2 illustrates the results of the sensitivity study conducted on the mesh seeding size, indicating that the FHD and peak von Mises stresses in the model converged for a mesh size of less than 2mm. The mesh convergence analysis was conducted using a single hip contact force at 30% stance phase of the gait cycle (maximum forces and stresses recorded).


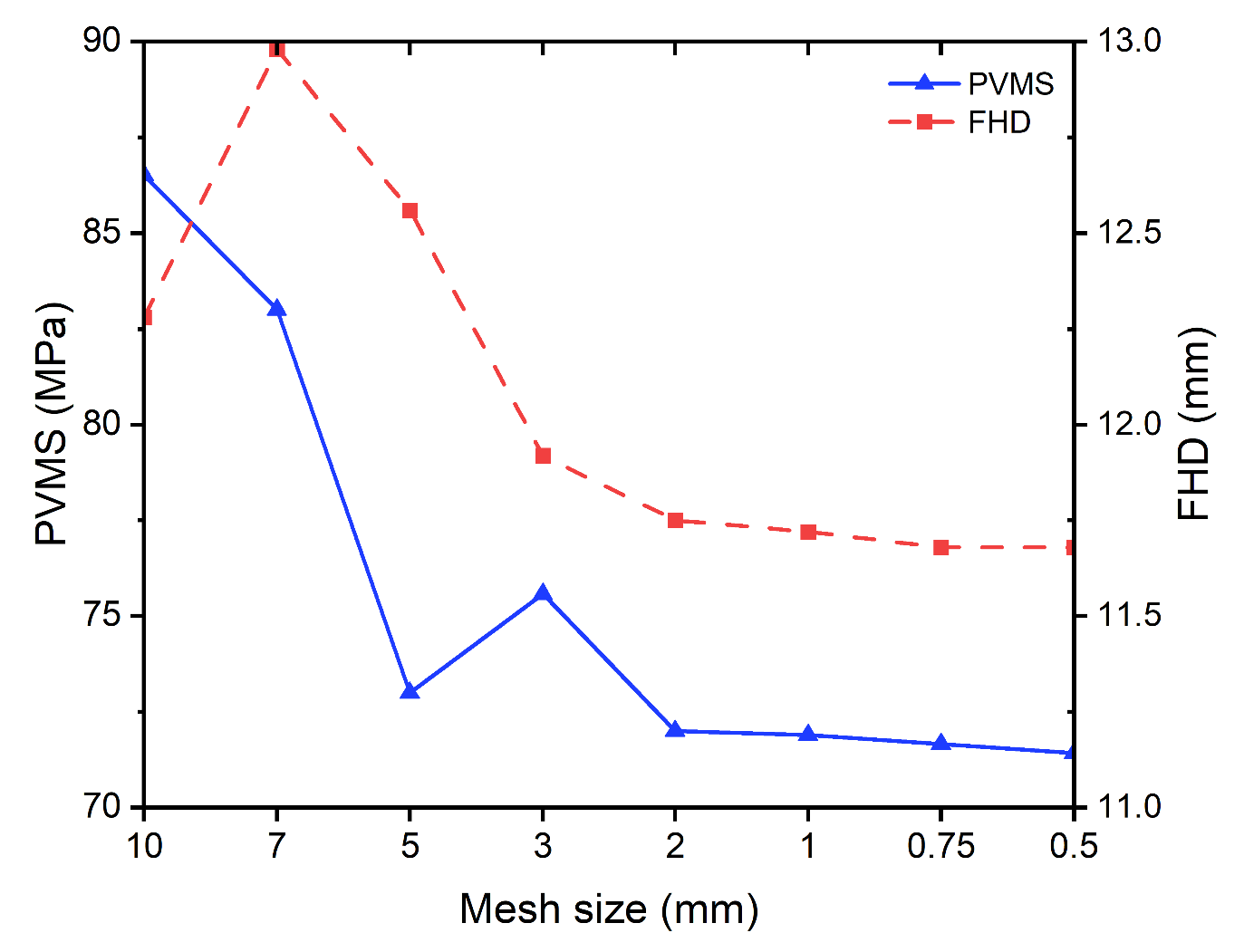


**Fig. S2**. Maximum FHD and peak von Mises stress (PVMS) results of the mesh size sensitivity study.

## Material properties sensitivity studies

Fig. S3 illustrates the results of the sensitivity study conducted on the material property set, indicating that FHD and peak Von Mises stresses in the model remained independent of the number of material property sets applied when the number of material sets is above ten. The material properties sensitivity analysis was conducted using a single hip contact force at 30% stance phase of the gait cycle (maximum forces and stresses recorded).


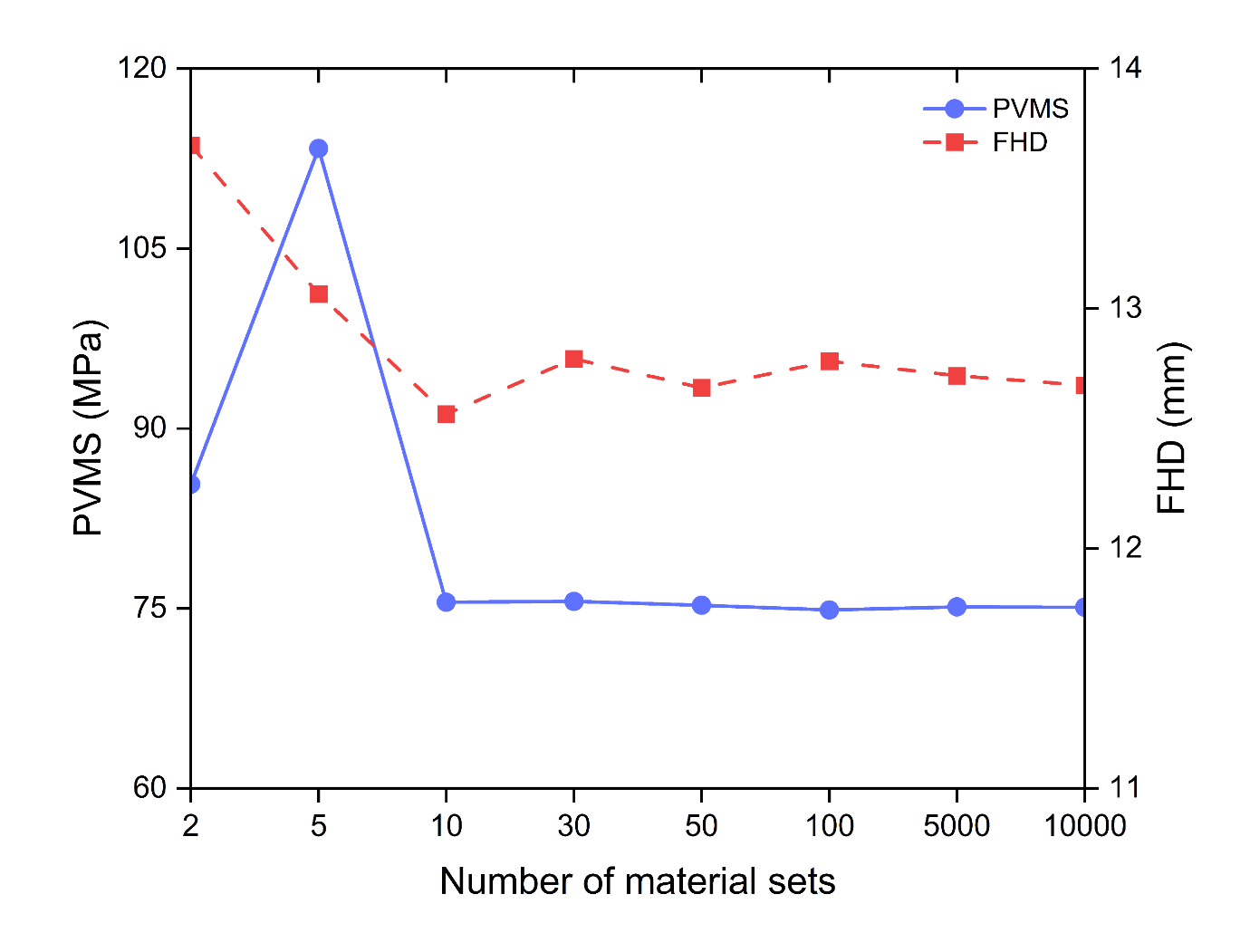


**Fig. S3**. Maximum FHD and peak von Mises stress (PVMS) results of the material property set sensitivy study.

# Statistical comparison

### Femoral head deflections

Table S3 shows the agreement in femoral head deflections (R^2^ and nRMSE) between the evaluated boundary conditions compared with the inertia relief method.

| **Model** | **Femoral head deflection** | **Fixed-knee** | | **Springs** | | **Isostatic** | | **Mid-shaft** | | **Biomechanical** | |
| --- | --- | --- | --- | --- | --- | --- | --- | --- | --- | --- | --- |
|  |  | R^2^ | nRMSE | R^2^ | nRMSE | R^2^ | nRMSE | R^2^ | nRMSE | R^2^ | nRMSE |
| **Normal** | Medial-lateral | 0.96 | 55.65 | 0.09 | 2.75 | 0.70 | 1.68 | 0.96 | 20.11 | 0.98 | 1.17 |
|  | Anterior-posterior | 0.29 | 22.46 | 0.26 | 15.59 | 0.29 | 1.16 | 0.09 | 5.20 | 0.82 | 1.83 |
|  | Superior-inferior | 0.96 | -7.65 | 0.93 | -0.93 | 0.94 | -0.86 | 0.97 | -2.58 | 0.96 | -0.29 |
|  | Resultant | 0.89 | 20.80 | 0.51 | 7.70 | 0.95 | 0.69 | 0.84 | 6.51 | 0.97 | 0.17 |
| **AVA -10°** | Medial-lateral | 0.92 | 86.03 | 0.01 | 5.03 | 0.64 | 1.82 | 0.92 | 30.48 | 0.91 | 2.70 |
|  | Anterior-posterior | 0.54 | 32.16 | 0.41 | 13.69 | 0.00 | 1.34 | 0.46 | 9.31 | 0.23 | 1.25 |
|  | Superior-inferior | 0.91 | -9.54 | 0.94 | -1.26 | 0.92 | -0.86 | 0.91 | -3.38 | 0.96 | -0.23 |
|  | Resultant | 0.93 | 24.86 | 0.68 | 7.87 | 0.91 | 0.72 | 0.89 | 7.70 | 0.90 | 0.27 |
| **AVA 45°** | Medial-lateral | 0.96 | 71.68 | 0.00 | 2.06 | 0.08 | 1.76 | 0.97 | 25.40 | 0.97 | 1.51 |
|  | Anterior-posterior | 0.07 | 37.88 | 0.05 | 46.20 | 0.65 | 1.05 | 0.24 | 10.68 | 0.42 | 7.48 |
|  | Superior-inferior | 0.94 | -7.64 | 0.92 | -0.78 | 0.98 | -0.92 | 0.97 | -2.65 | 0.96 | -1.11 |
|  | Resultant | 0.88 | 21.17 | 0.37 | 9.71 | 0.83 | 0.48 | 0.88 | 6.95 | 0.61 | 1.02 |
| **NSA 115°** | Medial-lateral | 0.88 | 88.82 | 0.22 | 3.81 | 0.65 | 2.24 | 0.88 | 33.61 | 0.37 | 3.81 |
|  | Anterior-posterior | 0.32 | 17.69 | 0.33 | 10.37 | 0.22 | 1.17 | 0.15 | 4.06 | 0.45 | 1.54 |
|  | Superior-inferior | 0.98 | -5.01 | 0.98 | -0.23 | 0.96 | -0.59 | 0.98 | -1.85 | 0.98 | -0.23 |
|  | Resultant | 0.96 | 16.17 | 0.65 | 4.14 | 1.00 | 0.58 | 0.97 | 5.31 | 0.75 | 0.32 |
| **NSA 150°** | Medial-lateral | 0.94 | 372.66 | 0.00 | 27.98 | 0.21 | 10.54 | 0.96 | 130.97 | 0.94 | 16.31 |
|  | Anterior-posterior | 0.45 | 41.71 | 0.49 | 21.57 | 0.02 | 1.36 | 0.33 | 11.95 | 0.70 | 2.11 |
|  | Superior-inferior | 0.81 | -51.59 | 0.89 | -9.05 | 0.90 | -4.19 | 0.85 | -17.64 | 0.96 | -2.21 |
|  | Resultant | 0.61 | 31.82 | 0.65 | 13.14 | 0.22 | 0.62 | 0.48 | 9.71 | 0.73 | 0.51 |

**Table S3.** R^2^ and nRMSE statistical values reported for femoral head deflections of the various models as compared to the inertia relief (IR) method.

### Peak von Mises stress

Table S4 presents the quantitative assessment of the agreement (R^2^ and nRMSE) between the evaluated boundary conditions and the inertia relief method for the peak von Mises stresses arising in the femur.

| **Model** | **Fixed-knee** | | **Springs** | | **Isostatic** | | **Mid-shaft** | | **Biomechanical** | |
| --- | --- | --- | --- | --- | --- | --- | --- | --- | --- | --- |
|  | R^2^ | nRMSE | R^2^ | nRMSE | R^2^ | nRMSE | R^2^ | nRMSE | R^2^ | nRMSE |
| **Normal** | 0.46 | 1.23 | 0.79 | 0.22 | 0.51 | 0.61 | 0.53 | 1.25 | 0.92 | 0.13 |
| **AVA -10°** | 0.59 | 1.59 | 0.68 | 0.25 | 0.56 | 0.48 | 0.52 | 1.67 | 0.97 | 0.09 |
| **AVA 45°** | 0.01 | 0.78 | 0.89 | 0.13 | 0.49 | 1.06 | 0.32 | 0.74 | 0.71 | 0.37 |
| **NSA 115°** | 0.83 | 1.30 | 0.91 | 0.31 | 0.86 | 0.27 | 0.78 | 1.34 | 0.98 | 0.13 |
| **NSA 150°** | 0.03 | 1.22 | 0.52 | 0.21 | 0.38 | 0.96 | 0.01 | 1.39 | 0.61 | 0.23 |

**Table S4.** R^2^ and nRMSE statistical values reported for peak von Mises Stress of the various models as compared to the inertia relief (IR) method.

# Bibliography

1. M. Heyland, A. Trepczynski, G. N. Duda, M. Zehn, K. D. Schaser, and S. Mardian, “Selecting boundary conditions in physiological strain analysis of the femur: Balanced loads, inertia relief method and follower load,” *Med Eng Phys,* vol. 37, no. 12, pp. 1180-5, Dec, 2015.
2. J. C. Ferré, R. Legoux, F. Marquet, C. Chevalier, J. L. Helary, J. P. Lumineau, A. Y. Le Cloarec, E. Orio, J. G. Barbin, and J. Y. Barbin, “A physico-mathematical model for the human femur, with and without a prosthesis, under the static constraints of one-legged stance,” *Surg. Radiol. Anat.,* vol. 9, no. 3, pp. 241-249, 1987.
3. A. D. Speirs, M. O. Heller, G. N. Duda, and W. R. Taylor, “Physiologically based boundary conditions in finite element modelling,” *J Biomech,* vol. 40, no. 10, pp. 2318-23, 2007.
4. T. Gang, L. Shilei, W. Dongmei, W. Gaofeng, and W. Chengtao, “Finite element analysis in femoral fixation with TA3 titanium compressioll plate,” *Adv Mat Res,* vol. 647, pp. 16-19, 2013.
5. R. Bayoglu, and A. F. Okyar, “Implementation of boundary conditions in modeling the femur is critical for the evaluation of distal intramedullary nailing,” *Med Eng Phys,* vol. 37, no. 11, pp. 1053-60, Nov, 2015.
6. K. Gok, and S. Inal, “Biomechanical comparison using finite element analysis of different screw configurations in the fixation of femoral neck fractures,” *Mech. Sci.,* vol. 6, no. 2, pp. 173-179, 2015.
7. J. J. Zhou, R. Yi, M. Zhao, D. Liu, R. F. Lv, W. T. Yu, and C. F. Du, “Personalized finite element modeling analysis of femur bone healing after intramedullary nailing,” *J. Mech. Med. Biol.,* vol. 16, no. 5, 2016.
8. T. Celik, I. Mutlu, A. Ozkan, and Y. Kisioglu, “The effect of cement on hip stem fixation: a biomechanical study,” *Australas. Phys. Eng. Sci. Med.,* vol. 40, no. 2, pp. 349-357, 2017.
9. K. Gok, S. Inal, A. Gok, and A. M. Pinar, “Biomechanical effects of three different configurations in Salter Harris type 3 distal femoral epiphyseal fractures,” *J. Braz. Soc. Mech. Sci. Eng.,* vol. 39, no. 4, pp. 1069-1077, 2017.
10. A. H. Abdullah, M. Todo, and Y. Nakashima, “Prediction of damage formation in hip arthroplasties by finite element analysis using computed tomography images,” *Med. Eng. Phys.,* vol. 44, pp. 8-15, 2017.
11. K. Gok, S. Inal, A. Gok, and E. Gulbandilar, “Comparison of effects of different screw materials in the triangle fixation of femoral neck fractures,” *J. Mater. Sci. Mater. Med.,* vol. 28, no. 5, 2017.
12. X. Chen, and Z. Mao, “Shape optimization of orthopedic fixation plate based on static stress analysis,” *MCB Mol. Cell. Biomech.,* vol. 15, no. 4, pp. 229-241, 2018.
13. K. N. Chethan, M. Zuber, S. N. Bhat, and S. B. Shenoy, “Comparative study of femur bone having different boundary conditions and bone structure using finite element method,” *Open Biomed. Eng. J.,* vol. 12, no. 1, pp. 115-134, 2018.
14. S. Inal, K. Gok, A. Gok, A. O. Uzumcugil, and S. N. Kuyubasi, “Should we really compress the fracture line in the treatment of Salter-Harris type 4 distal femoral fractures? A biomechanical study,” *J. Braz. Soc. Mech. Sci. Eng.,* vol. 40, no. 11, 2018.
15. K. Gok, S. Inal, L. Urtekin, and A. Gok, “Biomechanical performance using finite element analysis of different screw materials in the parallel screw fixation of Salter-Harris Type 4 fractures,” *J. Braz. Soc. Mech. Sci. Eng.,* vol. 41, no. 3, 2019.
16. D. Kluess, E. Soodmand, A. Lorenz, D. Pahr, M. Schwarze, R. Cichon, P. A. Varady, S. Herrmann, B. Buchmeier, C. Schroder, S. Lehner, and M. Kebbach, “A round-robin finite element analysis of human femur mechanics between seven participating laboratories with experimental validation,” *Comput. Methods Biomech. Biomed. Eng.,* vol. 22, no. 12, pp. 1020-1031, 2019.
17. K. Gok, S. Inal, and A. Gok, “Biomechanical Effects of Four Different Configurations In Salter Harris Type 4 Distal Femoral Epiphyseal Fractures,” *Politeknik Dergisi,* vol. 23, no. 1, pp. 151-159, 2020.
18. J. B. R. Rose, “Computational and experimental investigation on the effect of failure stress in a femur bone,” *J. Comput. Methods Sci. Eng.,* vol. 20, no. 1, pp. 315-330, 2020.
19. S. Rathor, J. Jena, R. Uddanwadikar, and A. Apte, "Finite Element Analysis of Type I and Type II Fracture with PFN Implant—A Comparative Study," 2021, pp. 243-251.
20. V. Kumar, A. R. Bakhtari, P. Himanshu, and W. Akhtar, "Comparative Analysis of Femur Bone’s Compatible Materials by Finite Element Analysis (FEA) Tool," 2021, pp. 507-516.
21. Z. Altai, E. Montefiori, B. van Veen, M. A. Paggiosi, E. V. McCloskey, M. Viceconti, C. Mazza, and X. S. Li, “Femoral neck strain prediction during level walking using a combined musculoskeletal and finite element model approach,” *PLoS One,* vol. 16, no. 2, 2021.
22. A. Gee, H. Bougherara, E. H. Schemitsch, and R. Zdero, “Biomechanical design using in-vitro finite element modeling of distal femur fracture plates made from semi-rigid materials versus traditional metals for post-operative toe-touch weight-bearing,” *Med. Eng. Phys.,* vol. 87, pp. 95-103, 2021.
23. T. Joshi, R. Sharma, V. Mittal, V. Gupta, and G. Krishan, “Dynamic Fatigue Behavior of Hip Joint under Patient Specific Loadings,” *Int. J. Automot. Mech. Eng.,* vol. 19, no. 3, pp. 10014-10027, 2022.
24. C. Fritz, L. Fischer, E. Wund, and M. F. Zaeh, “Inner design of artificial test bones for biomechanical investigations using topology optimization,” *Prog. Addit. Manuf.*, 2022.
25. M. O. Heller, "Finite element analysis in orthopedic biomechanics," pp. 637-658, 2022.
26. A. T. M. Phillips, “The femur as a musculo-skeletal construct: A free boundary condition modelling approach,” *Med. Eng. Phys.,* vol. 31, no. 6, pp. 673-680, 2009.
27. K. Frydrysek, T. Halo, D. Cepica, V. Machalla, K. Simeckova, O. Skoupy, R. Madeja, M. Havlicek, K. Dostalova, A. Trefil, L. Pleva, Z. Murcinkova, P. Krpec, and J. Hlinka, “Biomechanical Assessment of Cannulated Nails for the Treatment of Proximal Femur Fractures,” *Appl. Sci. (Basel),* vol. 12, no. 15, 2022.
28. U. Andreaus, and M. Colloca, “Prediction of micromotion initiation of an implanted femur under physiological loads and constraints using the finite element method,” *Proc Inst Mech Eng H* vol. 223, no. H5, pp. 589-605, 2009.
29. B. A. Behrens, I. Nolte, P. Wefstaedt, C. Stukenborg-Colsman, and A. Bouguecha, “Numerical investigations on the strain-adaptive bone remodelling in the periprosthetic femur: Influence of the boundary conditions,” *Biomed. Eng. Online,* vol. 8, 2009.
30. M. Lerch, A. Kurtz, C. Stukenborg-Colsman, I. Nolte, N. Weigel, A. Bouguecha, and B. A. Behrens, “Bone remodeling after total hip arthroplasty with a short stemmed metaphyseal loading implant: Finite element analysis validated by a prospective DEXA investigation,” *J. Orthop. Res.,* vol. 30, no. 11, pp. 1822-1829, 2012.
31. M. T. Bah, J. F. Shi, M. O. Heller, Y. Suchier, F. Lefebvre, P. Young, L. King, D. G. Dunlop, M. Boettcher, E. Draper, and M. Browne, “Inter-subject variability effects on the primary stability of a short cementless femoral stem,” *J. Biomech.,* vol. 48, no. 6, pp. 1032-1042, 2015.
32. I. T. Haider, P. Schneider, A. Michalski, and W. B. Edwards, “Influence of geometry on proximal femoral shaft strains: Implications for atypical femoral fracture,” *Bone,* vol. 110, pp. 295-303, 2018.
33. E. Reina-Romo, J. Rodriguez-Valles, and J. A. Sanz-Herrera, “In silico dynamic characterization of the femur: Physiological versus mechanical boundary conditions,” *Med. Eng. Phys.,* vol. 58, pp. 80-85, 2018.
34. M. Heyland, S. Checa, D. Kendoff, and G. N. Duda, “Anatomic grooved stem mitigates strain shielding compared to established total hip arthroplasty stem designs in finite-element models,” *Sci. Rep.,* vol. 9, 2019.
35. S. Jitprapaikulsarn, N. Chantarapanich, A. Gromprasit, C. Mahaisavariya, and C. Patamamongkonchai, “Single lag screw and reverse distal femur locking compression plate for concurrent cervicotrochanteric and shaft fractures of the femur: biomechanical study validated with a clinical series,” *Eur. J. Orthop. Surg. Traumatol.,* vol. 31, no. 6, pp. 1179-1192, 2021.
36. S. Jitprapaikulsarn, N. Chantarapanich, A. Gromprasit, C. Mahaisavariya, K. Sukha, and S. Chiawchan, “Dual plating for fixation failure of the distal femur: Finite element analysis and a clinical series,” *Med. Eng. Phys.,* vol. 111, 2023.
37. T. D. Szwedowski, W. R. Taylor, M. O. Heller, C. Perka, M. Muller, and G. N. Duda, “Generic Rules of Mechano-Regulation Combined with Subject Specific Loading Conditions Can Explain Bone Adaptation after THA,” *PLoS One,* vol. 7, no. 5, 2012.
38. K. C. Nithin Kumar, N. Griya, A. Shaikh, V. Chaudhry, and S. Chavadaki, “Structural analysis of femur bone to predict the suitable alternative material,” *Mater. Today: Proc.,* vol. 26, pp. 364-368, 2019.
39. D. Nolte, and A. M. J. Bull, “Femur finite element model instantiation from partial anatomies using statistical shape and appearance models,” *Med. Eng. Phys.,* vol. 67, pp. 55-65, 2019.
40. Y. C. Wei, K. S. Basaruddin, F. Mat, R. Daud, M. J. A. Safar, and T. D. Hoang, “Finite element analysis on femur subjected to knee joint forces during incline-decline walking,” *Int. J. Adv. Technol. Eng. Explor.,* vol. 9, no. 92, pp. 888-898, 2022.
41. A. D. Oza, N. Gupta, and R. Singh, “Design and non-linear finite element analysis of titanium-based femoral hip-stem for Indian population,” *Int. J. Interact. Des. Manuf.*, 2022.
42. J. V. Inacio, P. Schwarzenberg, R. S. Yoon, A. Kantzos, A. Malige, C. O. Nwachuku, and H. L. Dailey, “Boundary Conditions Matter-Impact of Test Setup on Inferred Construct Mechanics in Plated Distal Femur Osteotomies,” *J Biomech Eng,* vol. 144, no. 8, Aug 1, 2022.
